# Comparative Structural Modeling of Six Old Yellow Enzymes (OYEs) from the Necrotrophic Fungus *Ascochyta rabiei* : Insight into Novel OYE Classes with Differences in Cofactor Binding, Organization of Active Site Residues and Stereopreferences

**DOI:** 10.1371/journal.pone.0095989

**Published:** 2014-04-28

**Authors:** Shadab Nizam, Rajesh Kumar Gazara, Sandhya Verma, Kunal Singh, Praveen Kumar Verma

**Affiliations:** Plant Immunity Laboratory, National Institute of Plant Genome Research, Aruna Asaf Ali Marg, New Delhi, India; University of Florida, United States of America

## Abstract

Old Yellow Enzyme (OYE1) was the first flavin-dependent enzyme identified and characterized in detail by the entire range of physical techniques. Irrespective of this scrutiny, true physiological role of the enzyme remains a mystery. In a recent study, we systematically identified OYE proteins from various fungi and classified them into three classes viz. Class I, II and III. However, there is no information about the structural organization of Class III OYEs, eukaryotic Class II OYEs and Class I OYEs of filamentous fungi. *Ascochyta rabiei*, a filamentous phytopathogen which causes Ascochyta blight (AB) in chickpea possesses six OYEs (ArOYE1-6) belonging to the three OYE classes. Here we carried out comparative homology modeling of six ArOYEs representing all the three classes to get an in depth idea of structural and functional aspects of fungal OYEs. The predicted 3D structures of *A. rabiei* OYEs were refined and evaluated using various validation tools for their structural integrity. Analysis of FMN binding environment of Class III OYE revealed novel residues involved in interaction. The ligand *para*-hydroxybenzaldehyde (PHB) was docked into the active site of the enzymes and interacting residues were analyzed. We observed a unique active site organization of Class III OYE in comparison to Class I and II OYEs. Subsequently, analysis of stereopreference through structural features of ArOYEs was carried out, suggesting differences in R/S selectivity of these proteins. Therefore, our comparative modeling study provides insights into the FMN binding, active site organization and stereopreference of different classes of ArOYEs and indicates towards functional differences of these enzymes. This study provides the basis for future investigations towards the biochemical and functional characterization of these enigmatic enzymes.

## Introduction

Old Yellow Enzyme (OYE1) was initially isolated from the yeast *Saccharomyces pastorianus* by Warburg & Christian (1933) [1]. It was the first enzyme shown to possess a flavin cofactor, flavin mono-nucleotide (FMN). It has been well established that NAD(P)H serves as the physiological reductant for the enzyme-bound flavin [2], whereas several compounds such as quinines and many α/β-unsaturated aldehydes and ketones can act as oxidants [3]. Even though, majority of them are not naturally occurring. However, despite extensive characterization of this enzyme, the true physiological oxidant of OYE remains elusive till date. The gene encoding OYE1 was identified 58 years after the protein isolation [4]. Since then, a number of OYE homologs have been identified from other yeasts, bacteria, protists, plants and filamentous fungi [5]–[10]. Numerous metabolic functions for OYE homologs have been suggested including degradation of nitrate ester explosives in bacteria [11]–[13], oxidative stress response in yeasts [14]–[16], jasmonic acid biosynthesis in plants [17], and ergot alkaloid biosynthesis in filamentous fungi *Aspergillus fumigatus* and *Claviceps purpuria* [18]. Furthermore, biochemical characterizations of OYEs have revealed their potential to catalyze the stereoselective reduction of activated C = C bonds of structurally diverse α,β-unsaturated compounds [19]. These optically active reduced products include many commercially useful substrates for industrial applications [20]. Therefore, from the last few years, OYEs are being investigated as biocatalysts for the affordable production of a variety of biotechnological and pharmaceutical compounds.

Several OYE homologs from yeasts, bacteria and plants have been crystallized and their structures have been resolved [21]–[23]. All of these OYEs were shown to fold into a (β/α)_8_ barrel (or TIM barrel) with the FMN binding within the barrel near the carboxy-terminus of the β-sheet. Despite having a conserved overall structure, mechanistic differences and variation in substrate preference occur between the OYE family members. On the basis of distinct sequence and structural features, Toogood et al., divided OYEs into two classes [24]. The first class includes the well described and investigated members such as OYE1 from *Saccharomyces pastorianus* [25], 12-oxophytodienoate reductase from plants [26] and morphinone reductase from bacteria [27] and thus was named as classical OYEs. However, structures of only two fungal OYEs (from *S. pastorianus* and *Pichia stipitis*, members of *Saccharomycetes*), belonging to this class have been solved so far [25], [28]. The second class was named as thermophilic-like OYEs and includes reductases such as YqjM from *Bacillus subtilis* [29], PpOYE (XenA) from *Pseudomonas putida 86* [30] as well as TsOYE from *Thermus scotoductus SA-01* [31]. Thermophilic-like OYEs are limited to bacteria only and structure of this OYE class has not been reported from eukaryotes. Members of classical and thermophilic-like OYEs show notable structural differences. Thermophilic-like OYEs posses a unique shared active site composition which is not observed in the active sites of classical OYEs. In thermophilic-like OYEs, an arginine [29] or tryptophan finger [30] protrudes from one monomer into the active site of the adjacent monomer. Therefore, thermophilic-like OYEs are mostly tetrameric with few dimeric proteins, whereas classical OYEs are mostly dimeric with certain monomeric OYEs [49].

Recently, to gain some insight regarding the distribution of OYEs and their physiological function in fungi, we carried out a comprehensive genome-wide identification of OYE proteins in 60 fungal species [32]. On the basis of active site residues and phylogeny, the identified OYEs were classified into three classes. Our study not only showed the existence of thermophilic-like OYEs in the genomes of several fungi but also suggested the presence of a novel OYE class, in addition to the classical OYEs. Therefore, we named the classical OYEs as Class I, thermophilic-like OYEs as Class II and novel class OYEs as Class III. Interestingly, it was observed that majority of fungal species (39 out of 60 species) analyzed in this study, posses all the three OYE classes in their genomes. One of such fungal species is *Ascochyta rabiei*, the causal agent of Ascochyta blight (AB) in chickpea worldwide. Recent studies related to genome sequencing of this phytopathogen in our laboratory revealed six OYEs (ArOYE1-6) of which ArOYE1-3 are Class I, ArOYE4 and ArOYE5 are Class II and ArOYE6 is Class III member. However, the structural organization regarding FMN binding and active site organization of Class III OYEs, eukaryotic Class II OYEs and Class I OYEs of filamentous fungi have not been studied yet.

In the present study, homology models of six *A. rabiei* OYEs were generated in order to get an in depth idea of their structural and functional aspects. The predicted structures were refined by taking advantages of MODELLER and energy minimization, and evaluated by PROCHECK, ProSA and QMEAN to analyze their structural integrity. Each 3D model was compared with the representative member of the respective OYE Class. Subsequently, cofactor binding environment of each ArOYE was examined. The ligand *para*-hydroxybenzaldehyde (PHB) was docked into the models of each ArOYE and its interactions with the active site residues were analysed. Furthermore, structural features responsible for stereopreference regarding R/S selectivity of each ArOYE were analyzed. Our study for the first time provides new insights towards the structural organization of novel OYE classes and indicates differences in substrate specificity and possible function.

## Results

### Sequence comparison and alignment of ArOYEs

**Figure 1:**
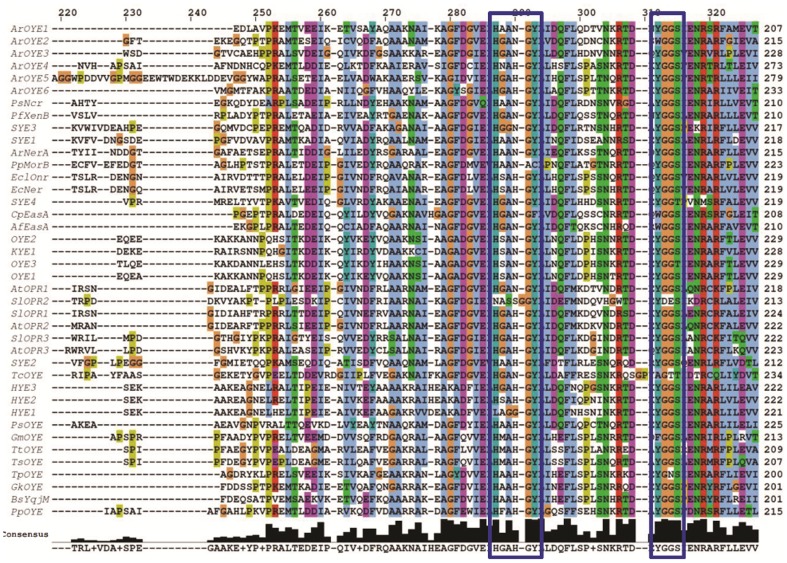
Multiple sequence alignment of ArOYE1-6 along with previously reported OYEs. The alignment includes OYEs from bacteria [*Pseudomonas syringae* (PsNcr) AAD16106.1, *Pseudomonas fluorescens* (PfXenB) AAF02539.1, *Shewanella oneidensis* (SYE1) NP_718044.1, (SYE2) NP_718043.1, (SYE3) NP_719682.1, (SYE4) NP_718946.1, *Agrobacterium radiobacter* (ArNerA) CAA74280.1, *Pseudomonas putida* (PpmorB) AAC43569.1, (PpOYE) NP_743414.1, *Enterobacter cloacae* (EclOnr) AAB38683.1, *Escherichia coli* (EcNer) NP_416167.1, *Geobacter metallireducens* (GmOYE) YP_006721534.1, *Thermus thermophilus* (TtOYE) YP_143423.1, *Thermus scotoductus* (TsOYE) YP_004203660.1, *Thermoanaerobacter pseudethanolicus* (TpOYE) YP_001664021.1, *Geobacillus kaustophilus* (GkOYE) YP_148185.1 and *Bacillus subtilis* (BsYqjM) NP_390263.1], yeast [*Saccharomyces cerevisiae* (OYE2) NP_012049.1, (OYE3) NP_015154.1, *Kluyveromyces lactis* (KYE) AAA98815.1, *Saccharomyces pastorianus* (OYE1) Q02899.3, *Hansenula polymorpha* (HYE1) AAN09952.1, (HYE2) AAN09953.1, (HYE3) AAN09954.1, and *Pichia stipitis* (PsOYE) XP_001384055.1], filamentous fungi [*Aspergillus fumigatus* (AfEasA) Q4WZ70.1 and *Claviceps purpurea* (CpEasA) AET79178.1], land plants [*Arabidopsis thaliana* (AtOPR1) CAA71627.1, (AtOPR2) NP_177795.1, (AtOPR3) NP_178662.1 and *Solanum lycopersicum* (SlOPR1) NP_001234781.1, (SlOPR2) NP_001233868.1, (SlOPR3) NP_001233873.1] and protozoa [*Trypanosoma cruzi* (TcOYE) AAA74448.1]. The multiple sequence and structure alignment program PROMALS3D was used to generate the alignment using default parameters. The positions of the conserved active sites are highlighted with the rectangular boxes. The consensus sequence is illustrated below the alignment.

To investigate the sequence conservation of ArOYEs, multiple sequence alignment was carried out. Full length sequences of ArOYEs were aligned using PROMALS3D program with default parameters. All the ArOYEs proteins varied moderately in their lengths (367–473 aa) as well as in positions of the conserved motifs. Sequence alignment suggested that there is less sequence conservation among the members (13–49% identity) and only the region containing the active site residues and the YGGS motif is well conserved among the six ArOYEs (Figure S1 in File S1). Comparing the sequences of members of same OYE class suggests more sequence similarity. Class I ArOYEs (ArOYE1-3) share 38–49% amino acid identity and Class II ArOYEs (ArOYE4 and ArOYE5) share 42% amino acid identity. In contrast, sequence identity of OYE proteins is fairly low between the members of Class I and Class II (19–26%), Class II and Class III (13–18%), and Class I and Class III (15–18%). The deduced amino acid sequences of ArOYE1-6 showed predicted molecular mass in the range of 40.6–51.5 kDa and theoretical pI in the range of 5.6–6.28 (Table 1). The analysis of ArOYE1-6 sequences in the conserved domain database (CDD) available at NCBI (http://www.ncbi.nlm.nih.gov/Structure/cdd/wrpsb.cgi) revealed interesting results. CDD predicted a conserved ‘OYE_like_FMN’ domain in ArOYE1-3 (Figure S2 in File S1). In contrast, it predicted ‘OYE_YqjM_FMN’ domain in ArOYE4 and ArOYE5, and ‘OYE_like_2_FMN’ domain in ArOYE6. To gain further insight, sequence alignment of ArOYEs was carried out with the previously known members. Sequence alignment suggested high sequence divergence among the homologs, sharing 11–91% of amino acid identity. The sequence conservation occurs among the core active site residues and the loop region containing the YGGS motif (Figure 1).

**Table 1:** Proteins used as templates for homology modeling of *A. rabiei* OYEs.

Target	MW	PI	Template	Template's Resolution (Å)	% Sequence identity	Description	Organism
ArOYE1	40.6	6.04	4K7Y	1.2	41	Old Yellow Enzyme (OYE1)	*Saccharomyces pastorianus*
ArOYE2	42.5	5.6	4K7Y	1.2	40	Old Yellow Enzyme (OYE1)	*Saccharomyces pastorianus*
ArOYE3	44.9	5.71	3P8I	1.19	43	Pentaerythritol tetranitrate reductase	*Enterobacter cloacae*
ArOYE4	46.5	6.28	3L5L	1.03	41	Xenobiotic reductase A (XenA)	*Pseudomonas putida*
ArOYE5	51.5	5.88	1Z41	1.3	35	Probable NADH-dependent flavin oxidoreductase (YqjM)	*Bacillus subtilis*
			3L5L	1.03	38	Xenobiotic reductase A (XenA)	*Pseudomonas putida*
ArOYE6	49.2	5.62	3KRU	1.6	29	NADH:flavin oxidoreductase/NADH oxidase	*Thermoanaerobacter pseudethanolicus*
			1ICP	1.9	31	12-oxophytodienoate reductase (OPR1)	*Solanum lycopersicum*

### Phylogenetic analysis of ArOYEs

**Figure 2:**
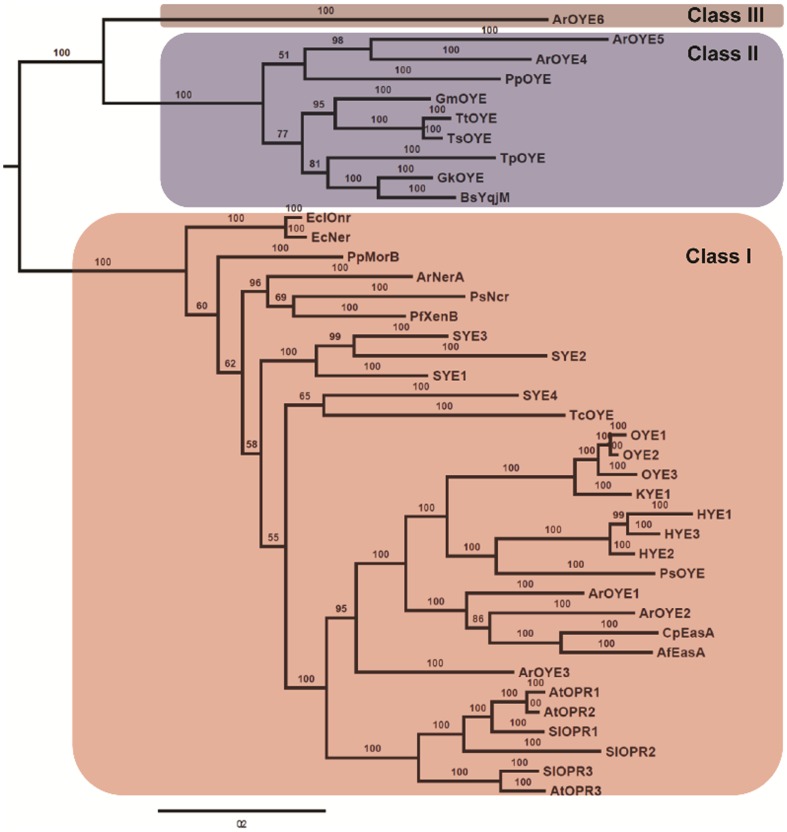
Evolutionary relationships of OYE family proteins. The multiple sequence alignment generated by PROMALS3D server was used to build the phylogenetic tree by Bayesian inference in MrBayes. The OYE proteins were classified into three distinct classes, designated as Class I, II, and III. Different colour was assigned to each class. The numbers at the nodes indicates the Bayesian posterior probabilities.

To investigate the evolutionary aspect of ArOYEs, phylogenetic analysis was carried out. It was conducted by the means of Bayesian inference (BI) using MrBayes (v3.2.2). Phylogenetic analysis indicated that six ArOYEs make two distinct clades, supported by robust posterior probabilities (100%) (Figure S3 in File S1). It was observed that ArOYE1, ArOYE2 and ArOYE3 make one clade, similarly ArOYE4, ArOYE5 and ArOYE6 made another clade. However, ArOYE4 and ArOYE5 were grouped together suggesting that they are more closely related and ArOYE6 is a distantly related member of the gene family (Figure S3 in File S1). To gain further insight regarding the evolution of ArOYEs, phylogenetic analysis was carried out with previously reported OYEs. This analysis indicated that six ArOYEs along with 34 previously known members make two distinct clades with robust branch support values (Figure 2). The first clade consists of Class I OYE from bacteria, yeasts, plants and filamentous fungi. The second clade consists of Class II OYEs along with ArOYE6. Further analysis of Class I OYEs revealed monophyletic origin of fungal and plant OYEs supported with high posterior probability (100%), indicating towards a common ancestor. Within the fungal subgroup, OYEs of yeasts are grouped together, whereas ArOYE1 and ArOYE2 are grouped along with OYEs of *A. fumigatus* and *C. purpuria*. Interestingly, ArOYE3 was found to be a distantly related member and wasn't grouped with any of the yeast or fungal OYEs. In contrast to plant and fungal OYEs, bacterial OYEs appeared paraphyletic, indicating towards diverse ancestors. Analysis of Class II OYEs revealed two subgroups. The first subgroup consists of bacterial OYEs and the second subgroup consists of ArOYE4 and ArOYE5 along with *Pseudomonas putida* OYE (PpOYE). This indicates a closer evolutionary relationship of ArOYE4 and ArOYE5 with PpOYE in comparison to other Class II OYE proteins. ArOYE6 was found as a distantly related member of all the OYEs analyzed further substantiating it to be a member of novel OYE class.

### Homology modeling, refinement and validation of ArOYEs

To study the structure of representative members of all the three OYE classes, homologs of OYE from *A. rabiei* were selected for homology modeling. The best templates of ArOYEs were selected through PSI-BLAST of each target protein against the PDB database. Single templates were used for each target protein except ArOYE5 and ArOYE6. Due to lack of crystal structure of Class III member from any organism, two proteins (TpOYE from *Thermoanaerobacter pseudethanolicus* and 12-oxophytodienoate reductase, SlOPR1 from *Solanum lycopersicum*) having moderate sequence identity with ArOYE6 were taken as templates. TpOYE belongs to Class II while SlOPR1 is a member of Class I OYE. Table 1 shows the proteins used as templates for homology modeling along with PDB IDs, resolution and their identity with the respective target protein. OYE domains of all the target proteins were modeled using MODELLER 9v11. Best models for each ArOYEs were chosen on the basis of their DOPE score. Using loop refinement protocol of MODELLER, initial refinement of each 3D model was carried out. The PROCHECK, ProSA and Qmean analyses were performed to assess the quality of the final structural models (values shown in Table S1 in File S1).

The Ramachandran plot showed the modeled domains of each ArOYE has more than 96% residues present in most favored and allowed regions (Figure S4a, Table S1 in File S1). Majority of the remaining residues (0.6–2.5%) were present in the generously allowed region, whereas only few residues (0–1.2%) were found in the disallowed region. The PROCHECK result summary showed the distribution of the main chain bond lengths (97.0–99.2%), bond angles (89.3–91.8) and planar groups (99.3–100%) present in all the modeled structures were within limits (Table S1 in File S1). Moreover, main-chain and side-chain parameters of each structure were in the better region. For a 3D model to be reliable, its goodness factor (G-factor) should be above −0.50. G-factor predicts the quality of overall bond and angle distances. The G-factor overall scores observed in ArOYE models were in the range of −0.25 to −0.11, suggesting reliable models of all the ArOYEs. The z-score calculated from ProSA-web server indicates the overall model quality and measures the deviation of the total energy of the structure with respect to an energy distribution derived from all experimental structures deposited in the Protein Data Bank (PDB). The z-scores of combined energy for modeled ArOYEs were negative and in the range of −8.61 to −6.13, suggesting the overall good quality of 3D structures (Figure S4b in File S1). Final confirmation was done by performing QMEAN analysis of the 3D models. QMEAN is a composite scoring function for homology models which estimates the quality of single model on the basis of the geometrical analysis. The QMEANnorm score and QMEAN Z-score of all the six ArOYEs suggest good quality models (Figure S4c, Table S1 in File S1). All the validated models were subjected to energy minimization using the GROMOS96 force field of Deep View. Finally, all the validated models were aligned with their respective templates, and their RMSD and TM-score were calculated using TM-Align. RMSD values of ArOYEs were in the range of 0.27 to 1.02 (Table S2 in File S1), suggesting the structural feature of each model is very close to its respective template.

### Characterization of homology model of ArOYEs

The 3D models of all the ArOYEs consist of eight β-sheets and eight α-helices (Figure S5 in File S1). All the 3D models comprised of one compact domain representing the frequently observed (β/α)_8_-barrel or TIM barrel fold, where a cylindrical core of eight twisted β-strands is surrounded by eight helices. Additional secondary structural elements occur on loops formed between the alternating sheet and helix core elements. Similar to other OYE homologs, all of the turns at the NH_2_-terminal end of the barrel are composed of only three or four residues, while the loops at the COOH-terminal end are much longer and build up the active sites. All ArOYE modeled structures, exhibit a characteristic short β-hairpin prior to helix α1 that closes the barrel at the N terminus. The overall structures of ArOYEs strongly resemble the structures of other OYE homologs (Figure S6 in File S1).

**Figure 3:**
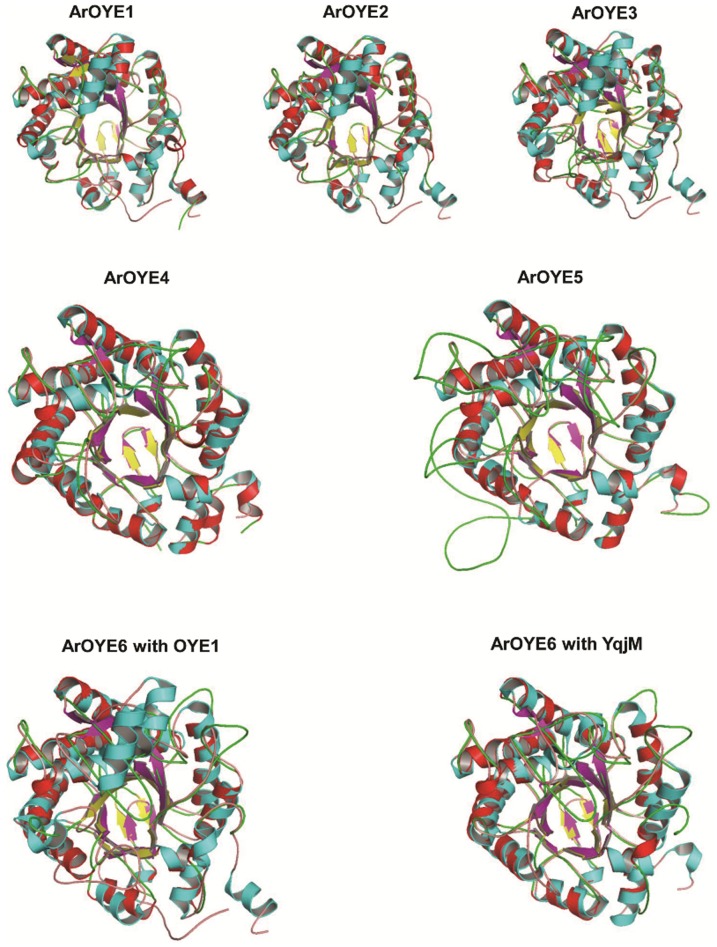
Comparison of ArOYEs with yeast OYE1 and YqjM. ArOYE1-3 (red and yellow) were superimposed upon yeast OYE1 (sky blue and dark blue), ArOYE4 and ArOYE5 (red and yellow) were superimposed upon *B. subtilis* YqjM (sky blue and dark blue), and ArOYE6 was superimposed upon both OYE1 and YqjM.

Comparing the predicted structures of ArOYE1-3 with OYE1, suggested their close structural resemblance (Figure 3). On the contrary, closer examination of the structures revealed differences. Although conformations of sheets and helices were in accordance with OYE1, the loops at the COOH-terminal end of the β-sheets of ArOYE1-3 adopted different conformations in the (β/α)_8_-barrel. The marked structural difference between ArOYE1-3 and OYE1 lies in the loop βL4. The loop βL4 contains the core active site residues viz. His^191^, Asn^194^, Tyr^196^ in case of OYE1. Although these residues are conserved in ArOYE1-3 as well but the length of the loop is different. The loop βL4 in OYE1 consists of 25 residues, whereas only 15 residues are present in ArOYE1-3. In order to get an insight about the structural conservation of ArOYE1-3, their 3D structures were superimposed upon each other (Figure S7a in File S1). All the three structures with eight α-helices and β-sheets along with secondary helices and sheets overlapped completely upon each other, suggesting these OYEs have conserved structural organization.

Since ArOYE4 and ArOYE5 are members of Class II, the predicted 3D structures were compared with the crystal structure of YqjM. As expected, the sheets and helices of both ArOYE4 and ArOYE5 were properly aligned with YqjM. In contrast, the loop regions of both ArOYEs showed different conformations from YqjM (Figure 3). The major structural difference in the monomers is in loop βL4, which consists of 25 residues in both ArOYE4 and ArOYE5. In contrast, 33 residues are present in YqjM. In addition, loop βL3 consists of 52 residues in ArOYE4, 73 residues in ArOYE5, and only 30 residues in YqjM. Another difference is in loop αL7 which is 4 and 5 residues in YqjM and ArOYE4, respectively. On the contrary, loop αL7 of ArOYE5 consists of 41 residues. This is evident from sequence alignment as well where a gap is introduced for proper alignment. However, this stretch is well conserved in corresponding OYE of closely related fungi. These analyses suggest ArOYE4 and ArOYE5 are slightly different in their overall geometry and points towards different enzymatic and molecular functions (Figure S7b in File S1).

Since Class I SlOPR1 and Class II TpOYE were used as templates for generating the 3D structure of ArOYE6, therefore we compared the modeled structure with both OYE1 and YqjM. Superimposing 3D structures of ArOYE6 with OYE1 suggested completely overlapping eight α-helices and β-sheets, however conformational differences were observed among the loop regions (Figure 3). The most notable difference was in the loop βL3. In ArOYE6, βL3 is 39 residues long with no secondary α-helices or β-sheets, whereas in OYE1 it is 48 residues long and contains two secondary α-helices and β-sheets. Superimposing the structure of ArOYE6 with YqjM suggested the proteins share more structural conservation (Figure 3). In contrast, we observed a notable difference between the two structures. The loop region βL4 is only 25 residues in ArOYE6, whereas it is 33 residues in YqjM. Taking together, structural comparisons indicate the overall structure of ArOYE6 resembling more with Class II proteins. In addition, it reveals the notable difference with Class II and thus indicates towards a novel structural organization of this class of fungal OYEs.

### FMN binding sites of ArOYEs

**Figure 4:**
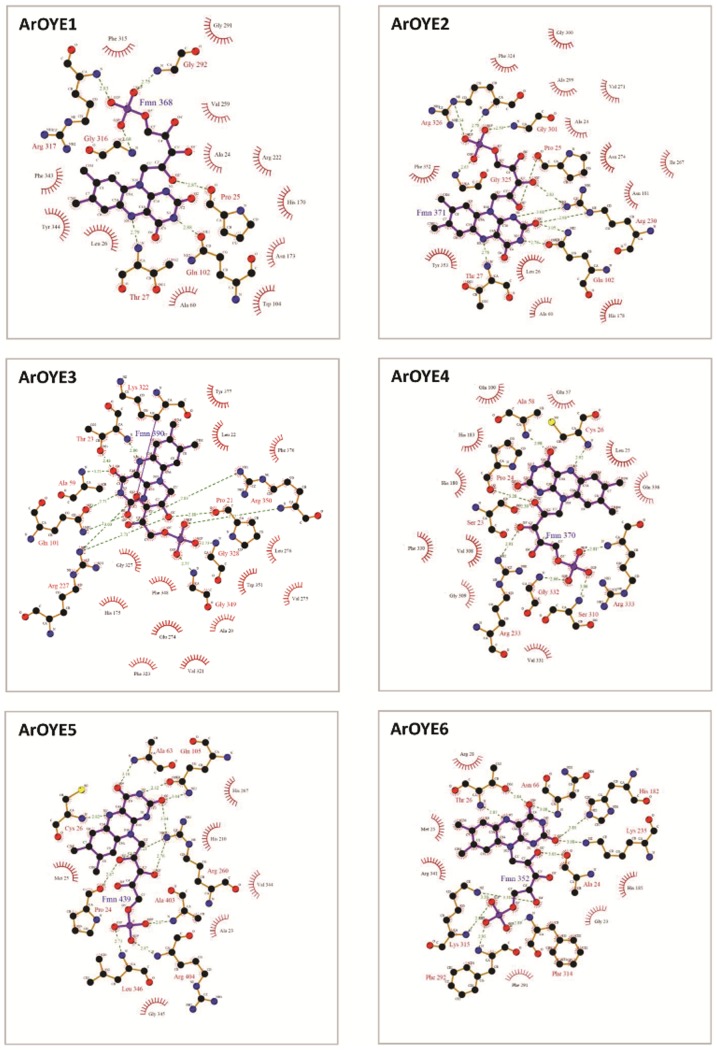
Analysis of FMN binding to the modeled ArOYEs. Ligplot diagrams of ArOYEs generated using PDBsum (44, 45), showing hydrogen bond as well as non bonded interactions.

To introduce FMN into the 3D structures of ArOYEs, MODELLER was used to supply restrains on the relative orientation of the FMN from respective templates to targets. FMN binding environment was then compared in a class-wise manner. From the crystal structure of OYE1, it has been deduced that Pro^35^, Thr^37^, Gly^72^, Gln^114^, Arg^243^, Gly^324^, Asn^325^, Phe^326^, Gly^345^, Gly^347^, and Arg^348^ contribute to the FMN binding sites [27]. Out of these eleven residues, seven residues (Pro^35^, Thr^37^, Gln^114^, Arg^243^, Gly^324^, Gly^347^ and Arg^348^, numbering according to OYE1) are conserved in ArOYE1-3. The residue, Phe^326^ is conserved in ArOYE1 and ArOYE3, whereas it is substituted with similar amino acid (Tyr) in ArOYE2. Similarly, Gly^72^ is conservatively substituted with (Ala) in all the three ArOYEs, and Gly^345^ is substituted with similar amino acid (Val) in ArOYE1 and (Ile) in ArOYE2 and ArOYE3. Only a single non-conservative amino acid substitution (Asn^325^ to Gly) was observed in all the three ArOYEs. In order to further confirm the FMN binding environment of these ArOYEs, LIGPLOT analysis was carried out. This analysis predicted the formation of hydrogen bond and/or hydrophobic interactions between these conserved residues and FMN (Figure 4). All the conserved and substituted residues were predicted to form hydrogen bond and/or hydrophobic interaction with FMN in ArOYE1-3 (Table S3 in File S1). Although the FMN binding residues are conserved among ArOYE1-3, their conformation is different in these proteins. Thus our analysis reveals similarity in the residues involved in FMN binding at the same time indicates differences in conformation of the FMN and interacting residues, pointing towards different substrate specificity among ArOYE1-3.

The crystal structure of YqjM has revealed that the residues Ser^23^, Pro^24^, Cys^26^, Ala^60^, Gln^102^, His^164^, His^167^, Arg^215^, Ser^249^, Gln^265^, Gly^284^, Met^285^, Phe^305^, Gly^307^, Arg^308^, Glu^309^ and Arg^312^ are involved in FMN binding [29]. Out of these seventeen residues, eleven residues (Pro^24^, Cys^26^, Ala^60^, Gln^102^, His^164^, His^167^, Arg^215^, Ser^249^, Gln^265^, Gly^284^, and Arg^308^, numbering according to YqjM) are conserved in both ArOYE4 and ArOYE5. Additionally, residues Ser^23^, Phe^305^ and Gly^307^ are conserved in ArOYE4, whereas they are substituted with similar amino acids Ala, Leu and Ala in ArOYE5. Further confirmation of the FMN binding environment of these ArOYEs was carried out as mentioned above. LIGPLOT prediction analysis revealed that out of the 17 residues of YqjM, 13 residues of ArOYE4 and 12 residues of ArOYE5 form hydrogen bond and hydrophobic interaction with FMN (Figure 4, Table S3 in File S1). Thus, the requirements for a functional FMN-binding site are fulfilled by the ArOYE4 and ArOYE5 proteins. Therefore, our sequence and structural analyses suggests that FMN-binding environment in ArOYE4 and ArOYE5 is at par with Class II OYEs.

In the absence of any known structure of Class III OYEs, model of ArOYE6 was directly taken for LIGPLOT analysis. The analysis indicated eight residues (Ala^38^, Thr^40^, Asn^80^, His^196^, Lys^249^, Phe^306^, Phe^328^, and Lys^329^, numbering according to ArOYE6) that form hydrogen bond and six residues (Gly^37^, Met^39^, Arg^42^, His^199^, Phe^305^ and Arg^355^) that form hydrophobic interaction with FMN (Figure 4, Table S3 in File S1). All the residues that form hydrogen bond are well conserved among the top 20 hits (Figure S8c in File S1), which we got from PSI-BLAST of ArOYE6 against the non-redundant protein database at NCBI. In addition, residues involved in the formation of hydrophobic interaction with FMN are also well conserved or substituted with similar amino acid residues. Comparing the residues of ArOYE6 that interact with FMN to that of other ArOYEs reveal vast difference in FMN binding environment among different classes of OYE. Therefore, our structural analysis for the first time reveals the FMN binding environment of ArOYE6 in particular and Class III OYEs in general.

### Active Site organization of ArOYEs

**Figure 5:**
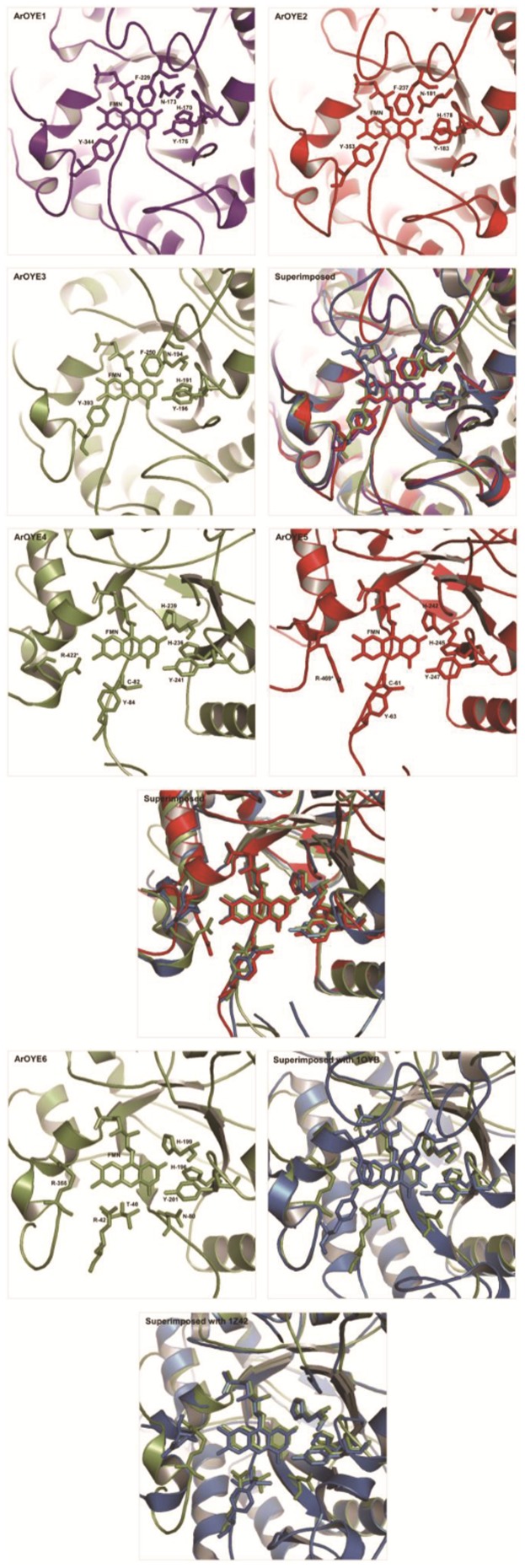
Characterization and comparison of the active sites of ArOYEs. The predicted active site residues were located in each ArOYE. Active site residue organization in ArOYE1-3 and their superimposition upon each other along with OYE1 (PDB ID: 1OYB, skyblue), in ArOYE4 and ArOYE5 and their superimposition upon each other along with YqjM (PDBID: 1Z42, sky blue), and in ArOYE6 and its superimposition upon OYE1 and YqjM.

To analyze the catalytic environment of ArOYEs, detailed analysis of the active site residues was carried out. The crystal structure of OYE1 substantiates that His^191^, Asn^194^, Tyr^196^, Phe^250^, Phe^296^ and Tyr^375^ contribute towards substrate binding [27]. Out of these six residues, five residues (His^191^, Asn^194^, Tyr^196^, Phe^250^ and Tyr^375^, numbering according to OYE1) are conserved in ArOYE1-3, whereas a residue corresponding to Phe^296^ in OYE1 is absent in all the three ArOYEs (Figure S8a in File S1). Therefore, the major difference between OYE1 and ArOYE1-3 in the catalytic region is the bigger size of the active site pocket in ArOYEs because of the absence of Phe^296^. Thus active sites of ArOYE1-3 appear to be more accessible to bulky substrates than that of OYE1. Comparing the active site residues side by side of ArOYE1-3 with OYE1 suggests similarities in the conformation of active site residues (Figure 5). Similarly, the crystal structure of YqjM has revealed that the residues Cys^26^, Tyr^28^, His^164^, His^167^, Tyr^169^ and Arg^336^ are involved in substrate binding [29]. All of these six residues are conserved in both ArOYE4 and ArOYE5 (Figure S8b in File S1). Comparing the catalytic region of ArOYE4 and ArOYE5 with YqjM suggested high similarities (Figure 5). This analysis authenticates that ArOYE4 and ArOYE5 are true eukaryotic homologs of YqjM.

Due to lack of crystal structure of Class III OYE, the true active site composition of this class is not known. To get idea regarding the active site of Class III, sequence comparison of ArOYE6 was carried out with Class I and Class II ArOYEs. Sequence alignment of ArOYE6 with other ArOYEs suggested that the core catalytic residues of OYE family (His^196^, His^199^, Tyr^201^, numbering according to ArOYE6) are also conserved in this protein (Figure 1). Additionally, sequence alignment was performed using ArOYE6 and its top 20 hits, which were obtained from a PSI-BLAST against the non-redundant protein database of NCBI with an E-value threshold of 10^−5^. Sequence alignment revealed that ArOYE6 shares high sequence identity (63–74%) with these proteins (Figure S8c in File S1). The core active site residues of ArOYE6 (His^196^, His^199^, Tyr^201^) are also well conserved among these proteins. However, alignment studies did not give any clue regarding the accessory residues involved in active site formation. Further analysis indicated the sequence conservation throughout the length of these proteins suggesting the possibilities of other conserved accessory residues involved in the formation of active site of Class III OYEs. Therefore, the 3D model of ArOYE6 was analyzed using active site prediction programs POOL and Q-SiteFinder. The predicted residues were validated through analyzing their position in the ArOYE6 structure (Figure 5). Only the residues making the active site pocket were selected. In this way following residues viz. Thr^40^, Arg^42^, Asn^80^, His^196^, His^199^, Tyr^201^ and Arg^355^ were selected as the predicted active site residues of ArOYE6. These residues are well conserved among the top 20 hits of ArOYE6 further demonstrating their role as active site residues (Figure S8c in File S1).

**Figure 6:**
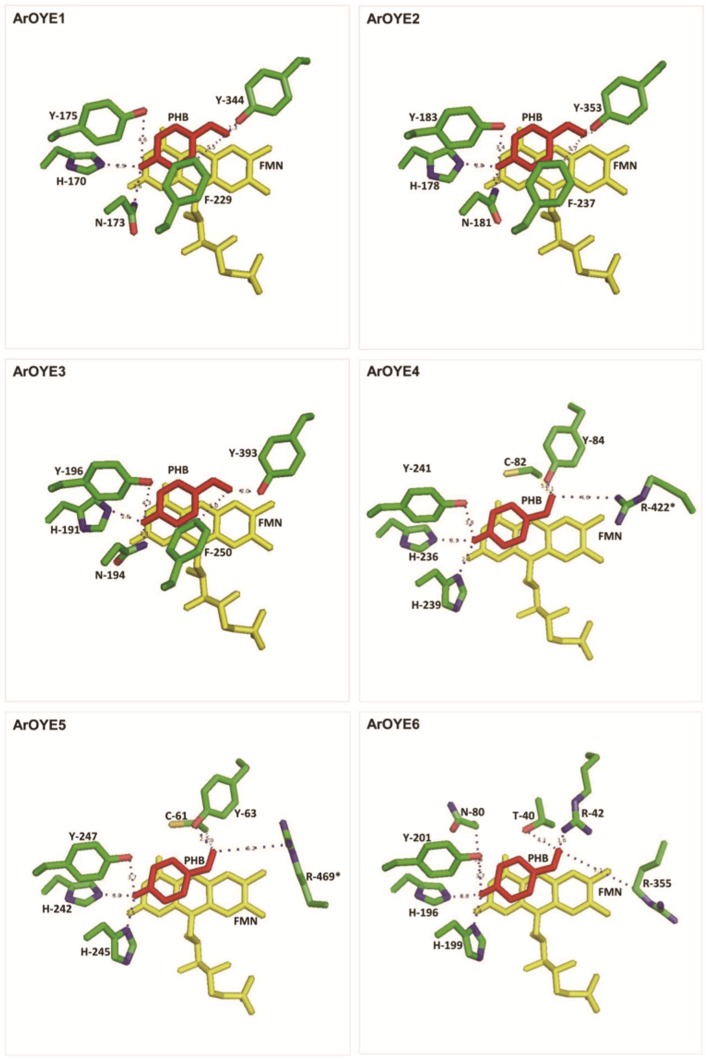
Docking of *para*-hydroxybenzyaldehyde (PHB) in the active sites of ArOYEs. *para*-hydroxybenzyaldehyde (PHB) was docked in the respective active site pocket of each ArOYE. Active site residues interacting with the ligand were analyzed. Position of active site residues in respective ArOYE is indicated by numbers.

In order to validate the active site residues of all ArOYEs, *para*-hydroxybenzyaldehyde (PHB) was docked into the active site pocket of each 3D model (Figure S9 in File S1). Interaction of PHB was analyzed with the predicted active site residues of each ArOYE (Figure 6). Close proximity of these residues with PHB substantiates their role in catalytic functions.

### Stereopreferences of ArOYEs

In recent years, it has been discovered that OYE family proteins carry out stereoselective reduction of activated C = C bonds [19]. In addition, to analyze the stereopreferences of OYEs, Oberdorfer et al. carried out extensive studies of structural features of exclusive *R* and *S* selective OYEs [33]. They observed a clear structure-specificity correlation and identified clusters on the basis of pseudo-atom distances. Their results clearly showed that in exclusive *R*-selective OYEs the pseudo-atom distance is >8 Å, whereas in exclusive *S*-selective OYEs the pseudo-atom distance is <7 Å. However, some special cases of OYEs were also observed, which showed intermediate distances (7.0–7.9 Å) and thus exhibit moderate stereospecificity.

**Figure 7:**
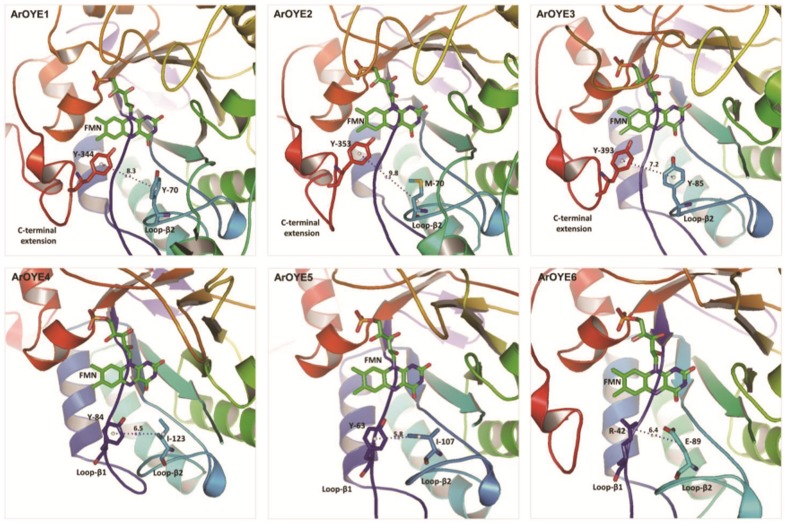
Stereopreference in ArOYEs. Pseudo-atoms were generated for the residues described by Oberdorfer et al. (33) involved in the stereoselectivity of ArOYEs. Bond distances between pseudo-atoms were analyzed for each ArOYE. Pseudo-atom distances indicate that ArOYE1 and ArOYE2 are exclusive *R*-selective, ArOYE3 is moderately selective and ArOYE4-6 are exclusive *S*-selective.

To analyze the stereopreferences of ArOYEs, we followed the structural features described by Oberdorfer et al. [33]. Corresponding residues involved in stereoselectivity were identified in each ArOYE through sequence and structure alignments. Pseudo-atoms were generated for the residue pair involved in stereoselectivity, in each ArOYEs. Analysis of pseudo-atom distances in ArOYEs revealed interesting results. The pseudo-atom distance is >8 Å in both ArOYE1 and ArOYE2, which indicates these proteins are exclusive *R*-selective (Figure 7). Interestingly, the pseudo-atom distance of ArOYE3 was 7.2 Å, suggesting moderate stereospecificity in this OYE. Similarly, in ArOYE4 and ArOYE5 the pseudo-atom distances were 6.5 Å and 5.8 Å, respectively thus indicating exclusive *S*-selectivity of these proteins (Figure 7). In the same way, the pseudo-atom distance of ArOYE6 was 6.4 Å, indicating this Class III OYE protein to be exclusive *S*-selective (Figure 7). Therefore, our results shows that ArOYEs have all the three types of stereopreferences from exclusive *R*-selective, moderate selective and exclusive *S*-selective.

## Discussion

Combinations of site-directed mutagenesis (SDM) and crystal structure studies have characterized the active site residues of OYE1 (OYE from *S. pastorianus*), which are well conserved across similar OYE proteins. A difference in active site organization was first observed when crystal structure of YqjM from *Bacillus subtilis* was resolved [29]. Thereafter, several homologs of YqjM have been isolated from other bacteria and their sequences were reported to posses the active site residues identical to YqjM. Few of these proteins were crystallized, which further confirmed the active site environment similar to that of YqjM. Thus a new class of OYE including YqjM and related bacterial proteins was discovered [24]. This class of OYE proteins contain an arginine or tryptophan finger, which protrudes from one monomer into the active site of the adjacent monomer. Thus displaying shared active site architecture. However, crystal structures of YqjM and related proteins have been reported only from bacteria and there is no structural information of eukaryotic homolog, till date. In order to gain some insight regarding the physiological function of OYEs in fungi, a comprehensive genome-wide identification of OYE proteins was carried out in 60 fungal species [32]. Active site residues and phylogeny were used to classify the identified OYEs into three classes viz. Class I, Class II and Class III. Class I OYEs posses the active site organization of OYE1, Class II OYEs contain the active site of YqjM, whereas Class III proteins appeared to have a unique active site organization. Thus our study showed the existence of YqjM like OYEs in the genomes of several fungi along with a novel OYE class, Class III. However, nothing is known about the active site organization or FMN binding environment of this class of proteins. In addition, there is no structural information about eukaryotic Class II OYEs and Class I OYEs of filamentous fungi. Therefore, due to lack of any experimental data, we decided to carry out *in silico* homology modeling of all the six OYEs of the chickpea blight fungus *Ascochyta rabiei* (ArOYE1-6), belonging to all the three OYE classes.

*In silico* homology modeling clearly indicated the overall structure of ArOYE1-3 resembles that of yeast OYE with few differences. Similarly, the structures of ArOYE4 and ArOYE showed the typical shared active site composition of YqjM class of OYE proteins. However, structure of ArOYE6 was quite different from the structures of OYE1 and YqjM, suggesting that it is a novel class of OYE protein. After determining and characterizing the structure of all the six ArOYEs, our next aim was to predict the FMN binding environment in these OYEs. Previous reports suggested that the cofactor FMN is non-covalently bound in the active site with the si-side of the alloxazine ring facing the solvent [25]–[29]. It is bound at the C-terminal end of the β-barrel, where loops βL1-βL8 set up the active-site cavity above the FMN. This binding environment is observed in both OYE1 and YqjM like proteins, however the residues involved are different between the two classes. In accordance with the previous reports, our study suggests that in all ArOYE models, FMN binds within the barrel near the carboxy-terminal ends of the β-strands in an extended conformation that lies roughly perpendicular to the barrel axis. In contrast, the residues involved in binding are different among the three OYE classes (Figure 4). Additionally, we observed difference in conformation among the members of the same class indicating towards different biochemical properties of ArOYEs. Active site predictions confirmed this hypothesis. Except for the core active site residues (His^196^, His^199^, Tyr^201^, residue numbering according to ArOYE6), the accessory residues are different in all the six ArOYEs. Accessory residues of Class I ArOYEs are identical to OYE1, whereas Class II ArOYEs posses accessory residues similar to YqjM. In contrast, the accessory residues of Class III OYE, ArOYE6 showed marked difference from Class I and Class II OYEs. On a broader way, the active site arrangement of ArOYE6 is somewhat related to Class II OYEs than Class I OYEs, suggesting that ArOYE6 is more closely related to Class II OYEs. On the other hand, accessory residues are different from members of both Class I and II proteins. The major differences with Class II proteins are substitution of Cys^26^ and Tyr^28^ of YqjM with Thr^40^ and Arg^42^ in ArOYE6 (Figure 5). Another important difference in ArOYE6 is the non-shared active site architecture. In Class II OYEs an arginine or tryptophan finger protrudes from one monomer into the active site of the adjacent monomer. In contrast, Arg^355^ comes from the same monomer in ArOYE6, therefore suggesting the monomeric nature of the enzyme.

In conclusion, using comparative homology modeling of six ArOYEs our study provides the first report about the structural analysis of fungal OYEs. Novel residues of Class III OYE, involved in interaction with FMN were revealed. In addition, active site residues of ArOYE6 was predicted and validated through docking of *para*-hydroxybenzaldehyde (PHB). In comparison to Class I and II OYEs our study reveals a unique active site organization of Class III OYEs. Furthermore, analysis of structural features involved in stereopreference of ArOYEs suggests differences in R/S selectivity of these OYEs. Future studies involving biochemical and molecular characterizations may further improve our understanding of the diverse enzymatic and molecular functions of these OYEs.

## Materials and Methods

### Sequence alignment and phylogenetic analysis

Multiple sequence and structure alignment program PROMALS3D server [34] was used to carry out protein sequence alignment of the full-length OYEs using default parameters. Sequence alignments were visualized using Jalview [35]. The phylogenetic relationships among OYE family members were determined by the means of Bayesian Markov Chain Monte Carlo (MCMC) inference of phylogeny as employed in MrBayes (v3.2.2) [36]. Two independent runs were performed using mixed amino acid substitution model where each run comprised 3,000,000 iterations, four simultaneous Markov Chain Monte Carlo (MCMC) chains and a sampling frequency of every 300 iterations with MCMC left at default settings. Tracer software (v1.5) (http://tree.bio.ed.ac.uk/software/tracer/) was employed to inspect convergence of the runs by analyzing the trace files generated by the Bayesian MCMC runs. Finally, a single tree was generated from the sampled trees obtained from independent runs by using the Sumt function of MrBayes. FigTree (v1.4.) (http://tree.bio.ed.ac.uk/software/figtree/) was used to visualize the phylogenetic trees.

### *In-silico* homology modeling of ArOYEs

In order to carry out homology modeling of ArOYEs, best templates were selected through PSI BLAST of each target protein against the PDB database (http://www.rcsb.org/pdb/home/home.do). The significant hits with >40% sequence identity and atomic resolution < 1.8 Angstrons, were selected as templates for each target protein except ArOYE5 and ArOYE6 where the templates taken were having <40% sequence identity. Table 1 shows the proteins used as templates for homology modeling along with PDB IDs, atomic resolution and their identity with the target protein. The three-dimensional structures of the target proteins were generated using a restrained-based approach in MODELLER9v11 [37]. For each OYE, 10 models were created and the one with the best score in terms of the discrete optimized protein energy (DOPE) potential implemented in MODELLER was chosen. Initial refinement of the 3D models generated was carried out with the help of loop refinement protocol of MODELLER. The assessment of the final structural models was carried out with PROCHECK [38], ProSA [39], [40] and QMEAN [41] analyses. The final deviation in the protein structure geometry was regularized by energy minimization with the GROMOS96 force field [42] using Deep View [43]. All the structures were visualized using PyMOL (http://www.pymol.org/).

### Analysis of FMN binding site

FMN was introduced into 3D structures through the restrains on the relative orientation of the cofactor from respective templates using MODELLER 9v11. FMN binding sites, in each ArOYEs were analyzed by generating LIGPLOT diagram using PDBsum [44], [45]. FMN interacting residues of each ArOYEs were categorized into two groups on the basis of type of interactions (Table S3 in File S1).

### Active site characterization

For the prediction of active site residues of ArOYEs, sequences of ArOYE1-5 were aligned with sequences of their respective templates using PROMALS3D. Subsequently, active site residues of all targets were identified by selecting the residues from alignments. Active sites of ArOYE6 was predicted using the POOL server [46] (http://www.pool.neu.edu) and Q-SiteFinder [47] (http://www.bioinformatics.leeds.ac.uk/qsitefinder).

### Ligand (substrate) structure

Structures of substrate *para*-hydroxybenzyaldehyde (PHB), which is known ligand of OYE proteins was obtained from NCBI PubChem. 2D structure was then sketched with ChemSketch tool (http://www.acdlabs.com/resources/freeware/chemsketch/) in MOL format then converted to PDB format using OpenBabel v2.3.1 (http://openbabel.org) for docking purpose.

### Ligand docking

The best 3D modeled structures of ArOYEs were utilized for docking using AutoDock4 [48]. Input structures of ArOYEs and PHB ligand were prepared by adding gasteiger charges and merging non-polar hydrogens using AutoDockTools. Map files were generated with AutoGrid4 using grid points 38 Å×44 Å×56 Å for ArOYE1, 40 Å×40 Å×40 Å for ArOYE2-5, and 62 Å×48 Å×50 Å for ArOYE6 with 0.375 Å spacing. Lamarckian Genetic Algorithm was used for simulations. The best docked ligand was chosen on the basis of lowest binding energy and conformation. Distances of interactions of ligand molecule with active site residues were analyzed using PyMOL (http://www.pymol.org/).

## Supporting Information

File S1 **Supplementary tables and figures.**
(PDF)
