# Supplementary material for: Comparative Structural Modeling of Six Old Yellow Enzymes (OYEs) from the Necrotrophic Fungus Ascochyta rabiei : Insight into Novel OYE Classes with Differences in Cofactor Binding, Organization of Active Site Residues and Stereopreferences
Source: PLoS One. 2014 Apr 28;9(4):e95989. doi: 10.1371/journal.pone.0095989 (PMC4002455; doi:10.1371/journal.pone.0095989)
Supplement: File S1 — Supplementary tables and figures. (PDF) [file pone.0095989.s001.pdf]

Table S1. PROCHECK, ProSA and QMEAN analysis of the final models of ArOYEs

Quality checks	Model					
	**ArOYE1**	**ArOYE2**	**ArOYE3**	**ArOYE4**	**ArOYE5**	**ArOYE6**
**PROCHECK**						
Most favored and allowed regions (%)	99.4	98.1	96.3	98.4	97.6	98.3
Generously allowed regions (%)	0.6	1.6	2.5	1.0	2.1	1.0
Disallowed regions (%)	0.0	0.3	1.2	0.6	0.3	0.7
**G-factor (overall)**	-0.19	-0.25	-0.25	-0.13	-0.22	-0.11
**M/c bond lengths**						
within limits (%)	98.0	97.0	97.0	98.1	98.0	99.2
highlighted (%)	2.0	3.0	3.0	1.9	2.0	0.8
**M/c bond angles**						
within limits (%)	89.3	89.6	89.4	91.4	89.4	91.8
highlighted (%)	10.7	10.4	10.6	8.6	10.6	8.2
**Planar groups**						
within limits (%)	100.0	100.0	99.3	100.0	99.4	100.0
highlighted (%)	0.0	0.0	0.7	0.0	0.0	0.0
**QMEAN**						
QMEANnorm score	0.72	0.69	0.69	0.72	0.69	0.59
QMEAN Z-Score	-0.54	-0.98	-0.92	-0.54	-0.92	-2.08
**ProSA**						
Z-Score	-8.61	-7.93	-6.91	-8.08	-6.13	-7.49

Table S2. RMSD and TM-scores of ArOYEs with their respective templates

ArOYEs	Template	Aligned length	RMSD	TM-score
ArOYE1	4K7Y	361	0.71	0.98611
ArOYE2	4K7Y	352	0.77	0.98744
ArOYE3	3P8I	352	0.27	0.99563
ArOYE4	3L5L	357	0.27	0.99846
ArOYE5	1Z41	336	0.67	0.99140
	3L5L	342	0.92	0.98547
ArOYE6	3KRU	309	1.02	0.90997
	1ICP	333	1.82	0.89966

Table S3. FMN binding residues of ArOYEs

**Bonds**	**ArOYE1**	**ArOYE2**	**ArOYE3**	**ArOYE4**	**ArOYE5**	**ArOYE6**
H Bond	Pro^{25}<br>Thr^{27}<br>Gln^{102}<br>Gly^{292}<br>Gly^{316}<br>Arg^{317}	Pro^{25}<br>Thr^{27}<br>Gln^{102}<br>Arg^{230}<br>Gly^{301}<br>Gly^{325}<br>Arg^{326}	Pro^{37}<br>Thr^{39}<br>Ala^{75}<br>Gln^{117}<br>Arg^{243}<br>Lys^{338}<br>Gly^{344}<br>Gly^{365}<br>Arg^{366}	Ser^{79}<br>Pro^{80}<br>Cys^{82}<br>Ala^{114}<br>Arg^{289}<br>Ser^{366}<br>Gly^{388}<br>Arg^{389}	Pro^{24}<br>Cys^{61}<br>Ala^{63}<br>Gln^{105}<br>Arg^{295}<br>Leu^{381}<br>Ala^{438}<br>Arg^{439}	Ala^{38}<br>Thr^{40}<br>Asn^{80}<br>His^{196}<br>Lys^{249}<br>Phe^{306}<br>Phe^{328}<br>Lys^{329}
Hydrophobic bond	Ala^{24}<br>Leu^{26}<br>Ala^{60}<br>Trp^{104}<br>His^{170}<br>Asn^{173}<br>Arg^{222}<br>Val^{259}<br>Ala^{290}<br>Gly^{291}<br>Phe^{315}<br>Phe^{343}<br>Tyr^{344}	Ala^{24}<br>Leu^{26}<br>Ala^{60}<br>His^{178}<br>Asn^{181}<br>Ile^{267}<br>Val^{271}<br>Asn^{274}<br>Ala^{299}<br>Gly^{300}<br>Phe^{324}<br>Phe^{352}<br>Tyr^{353}	Ala^{36}<br>Leu^{38}<br>His^{191}<br>Glu^{290}<br>Val^{291}<br>Leu^{292}<br>Val^{337}<br>Phe^{339}<br>Gly^{343}<br>Phe^{364}<br>Trp^{367}<br>Phe^{392}<br>Tyr^{393}	Leu^{81}<br>Glu^{113}<br>Gln^{156}<br>His^{236}<br>His^{239}<br>Val^{364}<br>Gly^{365}<br>Phe^{386}<br>Val^{387}<br>Gln^{392}	Ala^{58}<br>Met^{60}<br>His^{242}<br>His^{245}<br>Val^{379}<br>Gly^{380}	Gly^{37}<br>Met^{39}<br>Arg^{42}<br>His^{199}<br>Phe^{305}<br>Arg^{355}

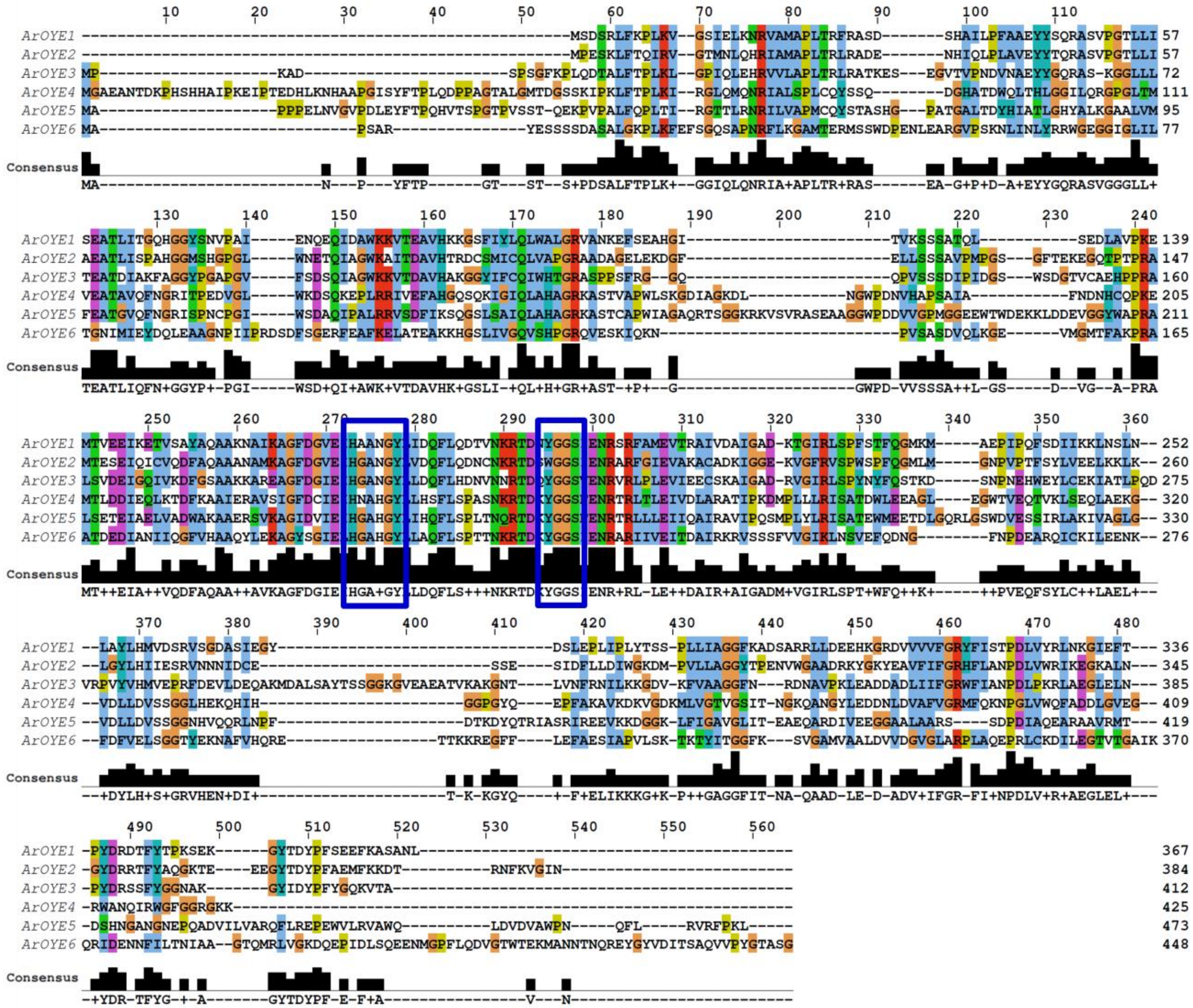

Figure S1. Multiple sequence alignment of full length ArOYE1-6 proteins. The alignment of ArOYEs viz. ArOYE1 (KF644454), ArOYE2 (KF644455), ArOYE3 (KF644456), ArOYE4 (KF644457), ArOYE5 (KF644458) and ArOYE6 (KF644459) was generated with the PROMALS3D server using default parameters. The positions of the core active site residues are highlighted with the rectangular boxes. The consensus sequence is illustrated below the alignment.

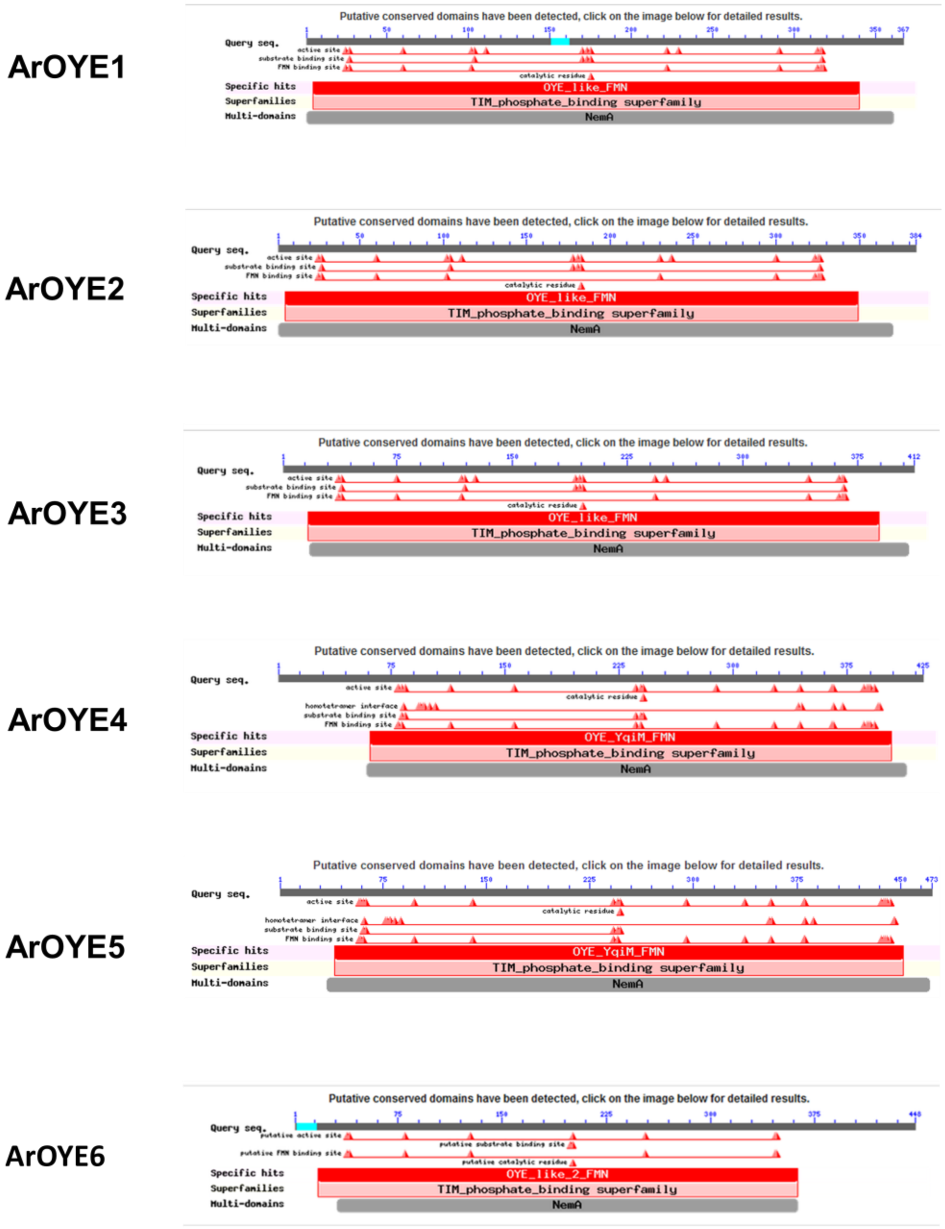

Figure S2. Conserved domains in ArOYEs.

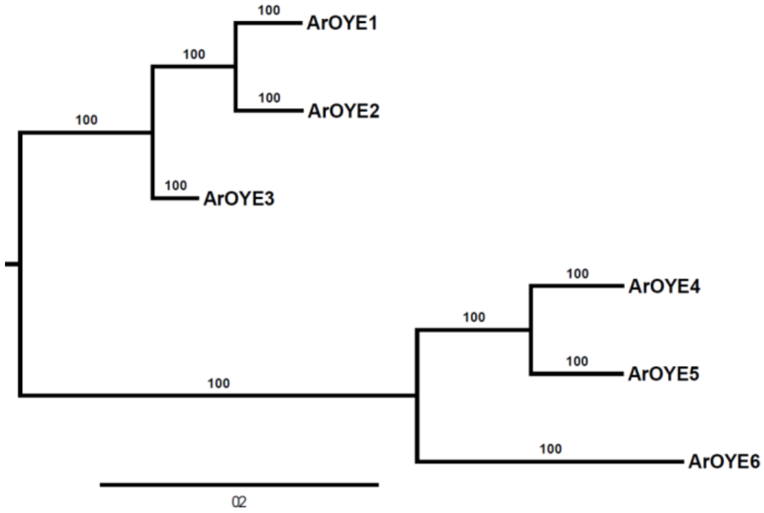

Figure S3. Evolutionary relationships of ArOYEs. The multiple sequence alignment of ArOYEs was generated by PROMALS3D server and was used to build the phylogenetic tree by Bayesian inference in MrBayes. The numbers at the nodes indicates the Bayesian posterior probabilities.

A

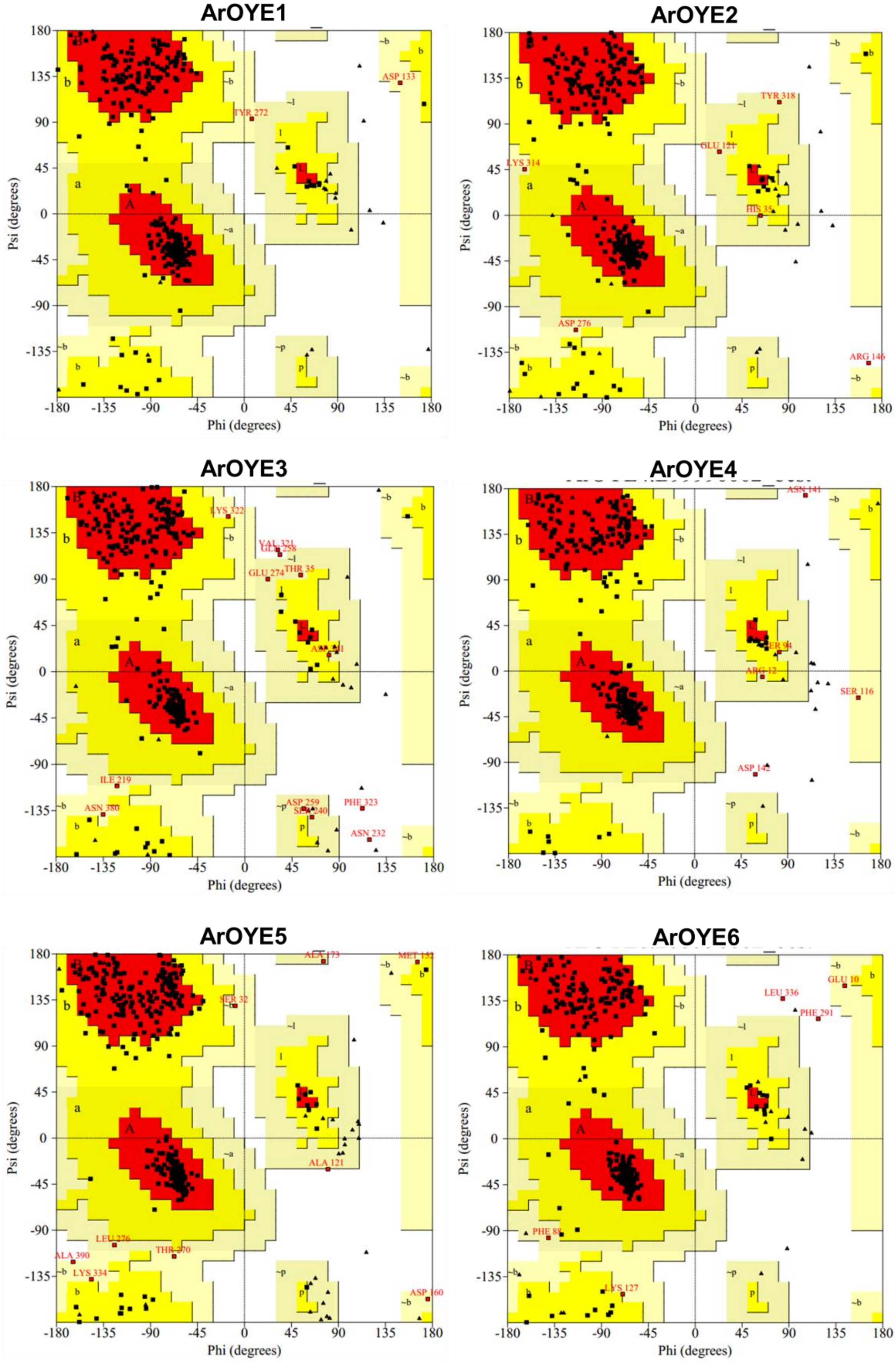
ArOYE1
ArOYE2
ArOYE3
ArOYE4
ArOYE5
ArOYE6
Psi (degrees)
Phi (degrees)
ASP 133
TYR 272
TYR 318
GLU 121
LYS 314
HIS 35
ASP 276
ARG 146
LYS 322
VAL 321
THR 35
GLU 274
ILE 219
ASN 380
ASP 259
PHE 323
ASN 232
ASN 141
SER 116
ARG 12
ASP 142
ALA 173
MET 132
SER 32
ALA 121
LEU 276
ALA 390
THR 270
LYS 334
ASP 160
LEU 336
GLU 10
PHE 291
PHE 88
LYS 127

B

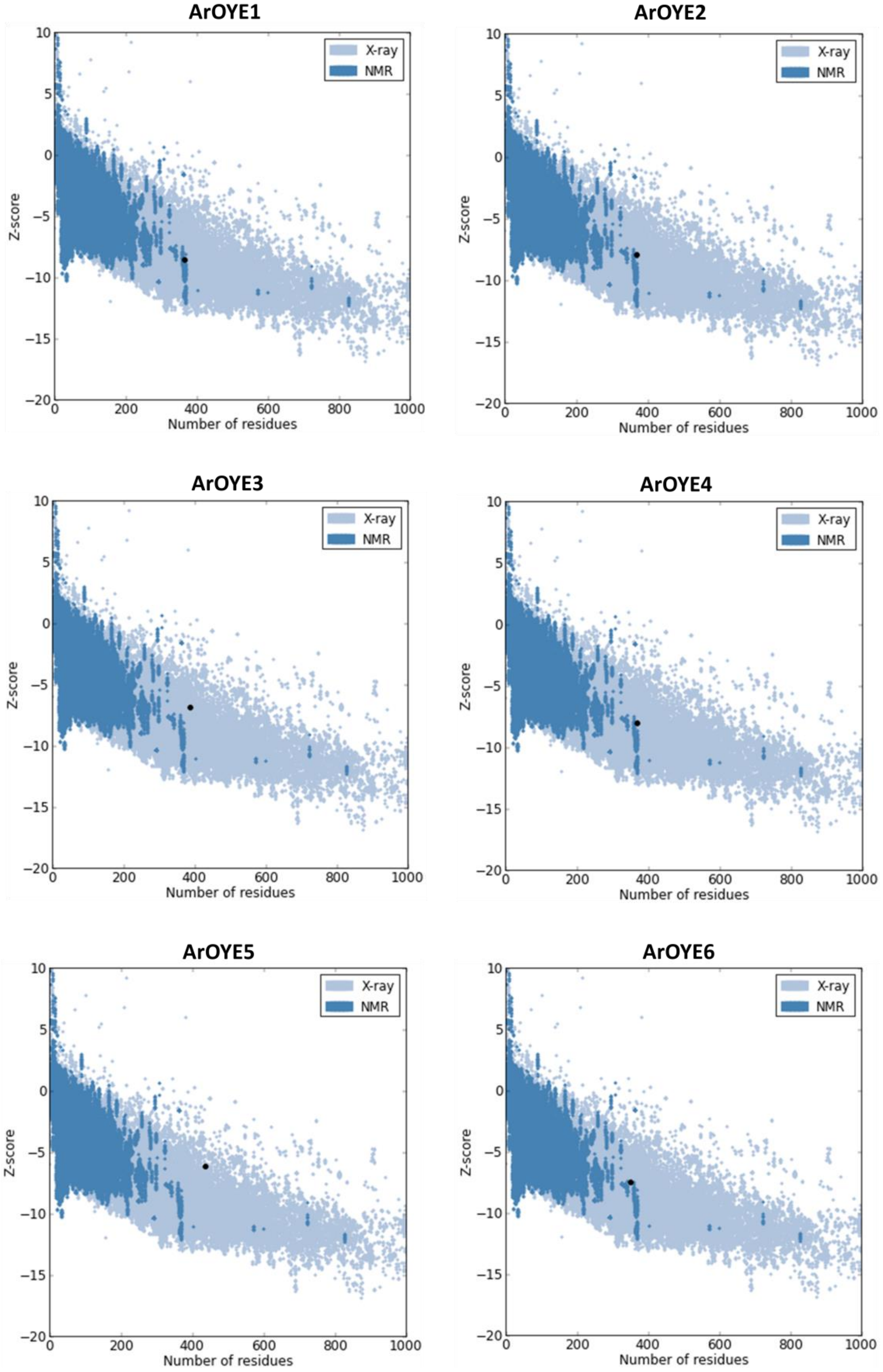

ArOYE1
ArOYE2
ArOYE3
ArOYE4
ArOYE5
ArOYE6
X-ray
NMR
Z-score
Number of residues
10
5
0
−5
−10
−15
−20
0
200
400
600
800
1000

C

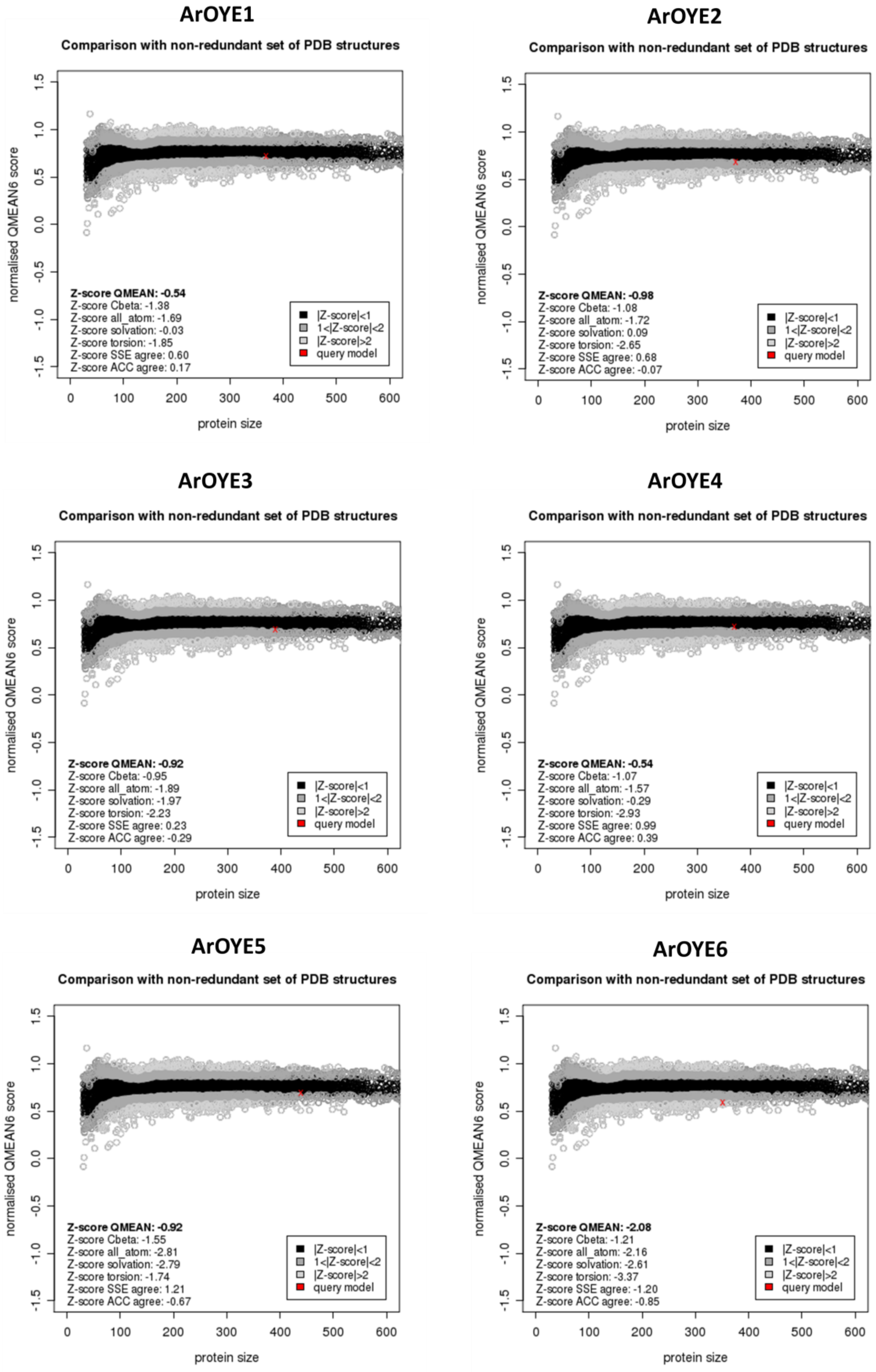
ArOYE1
Comparison with non-redundant set of PDB structures
normalised QMEAN6 score
protein size
Z-score QMEAN: -0.54
Z-score Cbeta: -1.38
Z-score all_atom: -1.69
Z-score solvation: -0.03
Z-score torsion: -1.85
Z-score SSE agree: 0.60
Z-score ACC agree: 0.17
|Z-score|<1
1<|Z-score|<2
|Z-score|>2
query model
ArOYE2
Comparison with non-redundant set of PDB structures
normalised QMEAN6 score
protein size
Z-score QMEAN: -0.98
Z-score Cbeta: -1.08
Z-score all_atom: -1.72
Z-score solvation: 0.09
Z-score torsion: -2.65
Z-score SSE agree: 0.68
Z-score ACC agree: -0.07
|Z-score|<1
1<|Z-score|<2
|Z-score|>2
query model
ArOYE3
Comparison with non-redundant set of PDB structures
normalised QMEAN6 score
protein size
Z-score QMEAN: -0.92
Z-score Cbeta: -0.95
Z-score all_atom: -1.89
Z-score solvation: -1.97
Z-score torsion: -2.23
Z-score SSE agree: 0.23
Z-score ACC agree: -0.29
|Z-score|<1
1<|Z-score|<2
|Z-score|>2
query model
ArOYE4
Comparison with non-redundant set of PDB structures
normalised QMEAN6 score
protein size
Z-score QMEAN: -0.54
Z-score Cbeta: -1.07
Z-score all_atom: -1.57
Z-score solvation: -0.29
Z-score torsion: -2.93
Z-score SSE agree: 0.99
Z-score ACC agree: 0.39
|Z-score|<1
1<|Z-score|<2
|Z-score|>2
query model
ArOYE5
Comparison with non-redundant set of PDB structures
normalised QMEAN6 score
protein size
Z-score QMEAN: -0.92
Z-score Cbeta: -1.55
Z-score all_atom: -2.81
Z-score solvation: -2.79
Z-score torsion: -1.74
Z-score SSE agree: 1.21
Z-score ACC agree: -0.67
|Z-score|<1
1<|Z-score|<2
|Z-score|>2
query model
ArOYE6
Comparison with non-redundant set of PDB structures
normalised QMEAN6 score
protein size
Z-score QMEAN: -2.08
Z-score Cbeta: -1.21
Z-score all_atom: -2.16
Z-score solvation: -2.61
Z-score torsion: -3.37
Z-score SSE agree: -1.20
Z-score ACC agree: -0.85
|Z-score|<1
1<|Z-score|<2
|Z-score|>2
query model

Figure S4. Evaluation of the modeled ArOYEs. (A) Ramachandran plot analysis of the best models of ArOYE1-6 protein. The plots clearly show that the majority of residues of each proteins falls under the allowed region. (B) ProSA-web plot analysis and (C) QMEAN Z-plot analysis, of the modeled ArOYEs

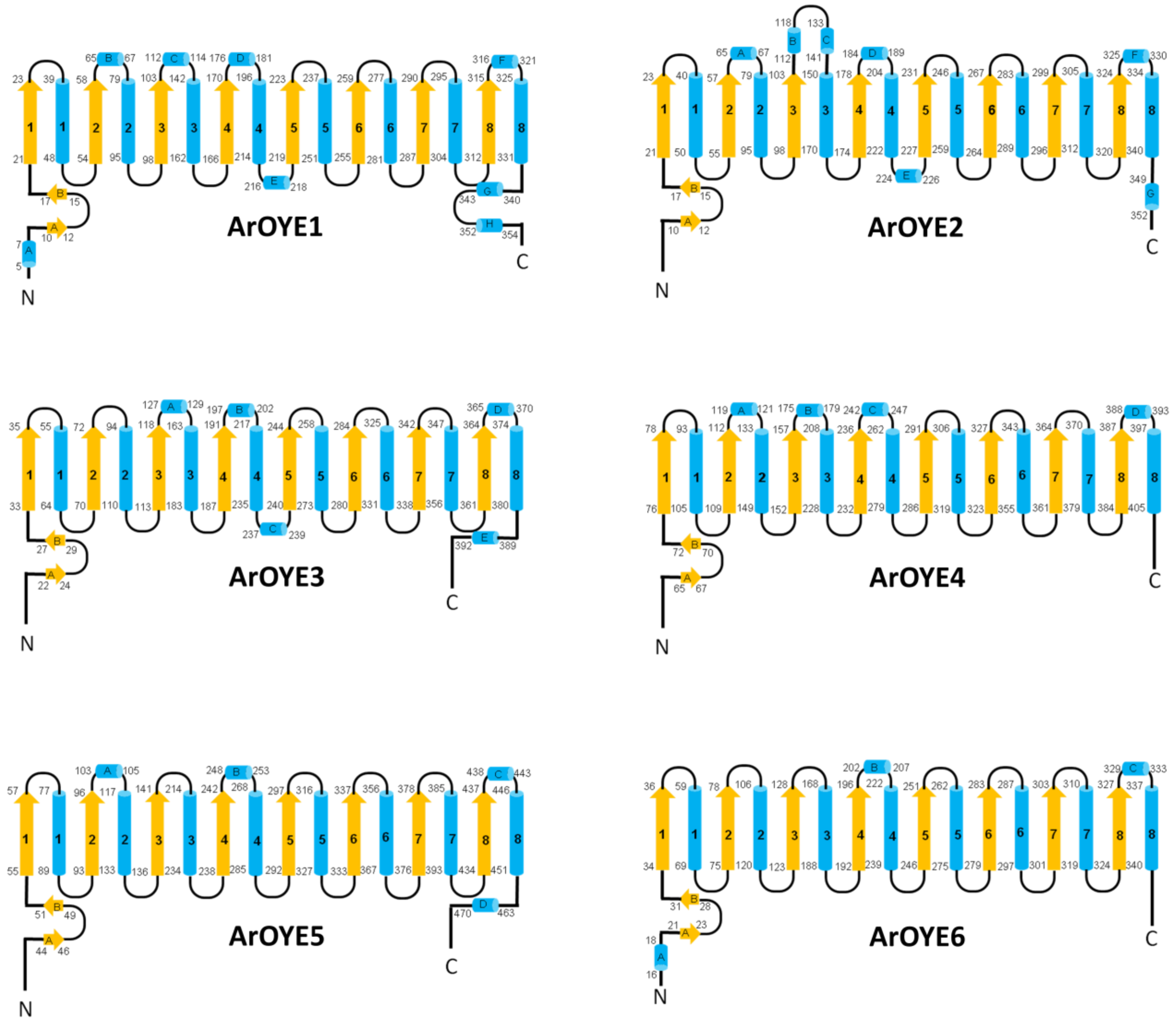

Figure S5. Secondary structure of ArOYEs. The yellow arrows indicate β sheets, blue cylinders indicate helix and the black lines indicate loops.

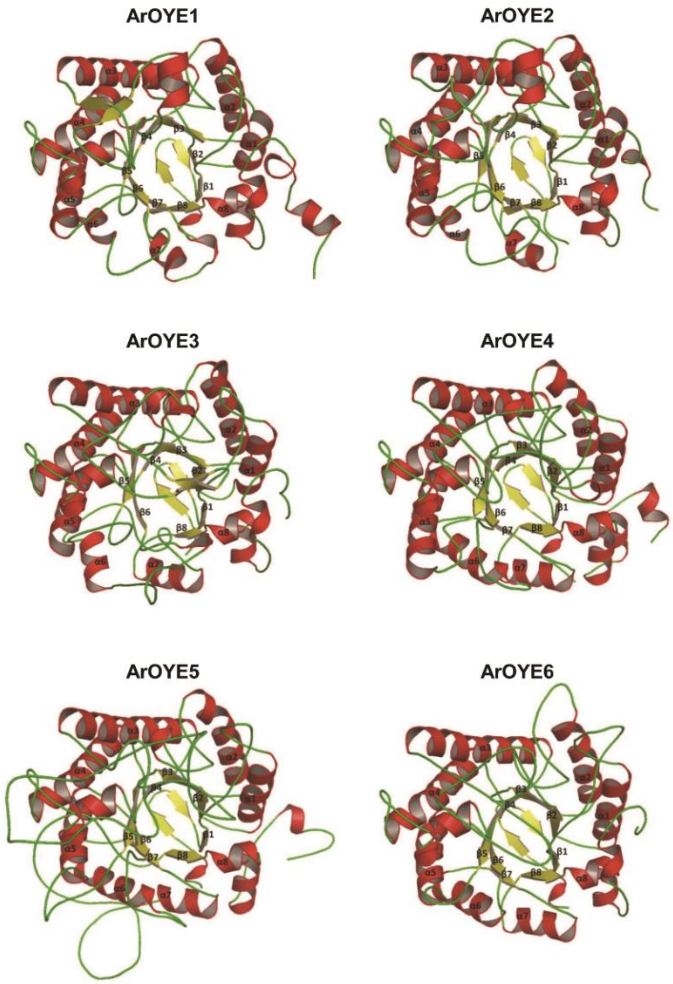

Figure S6. Modeled 3D structures of ArOYEs. The ribbon diagram representation of the 3D modeled structures. The alpha helices are displayed in red colour, sheets are displayed in yellow and loops are displayed in green. All the ArOYEs posses the alternate eight β-sheets and alpha helices, forming the characteristic TIM barrel structure.

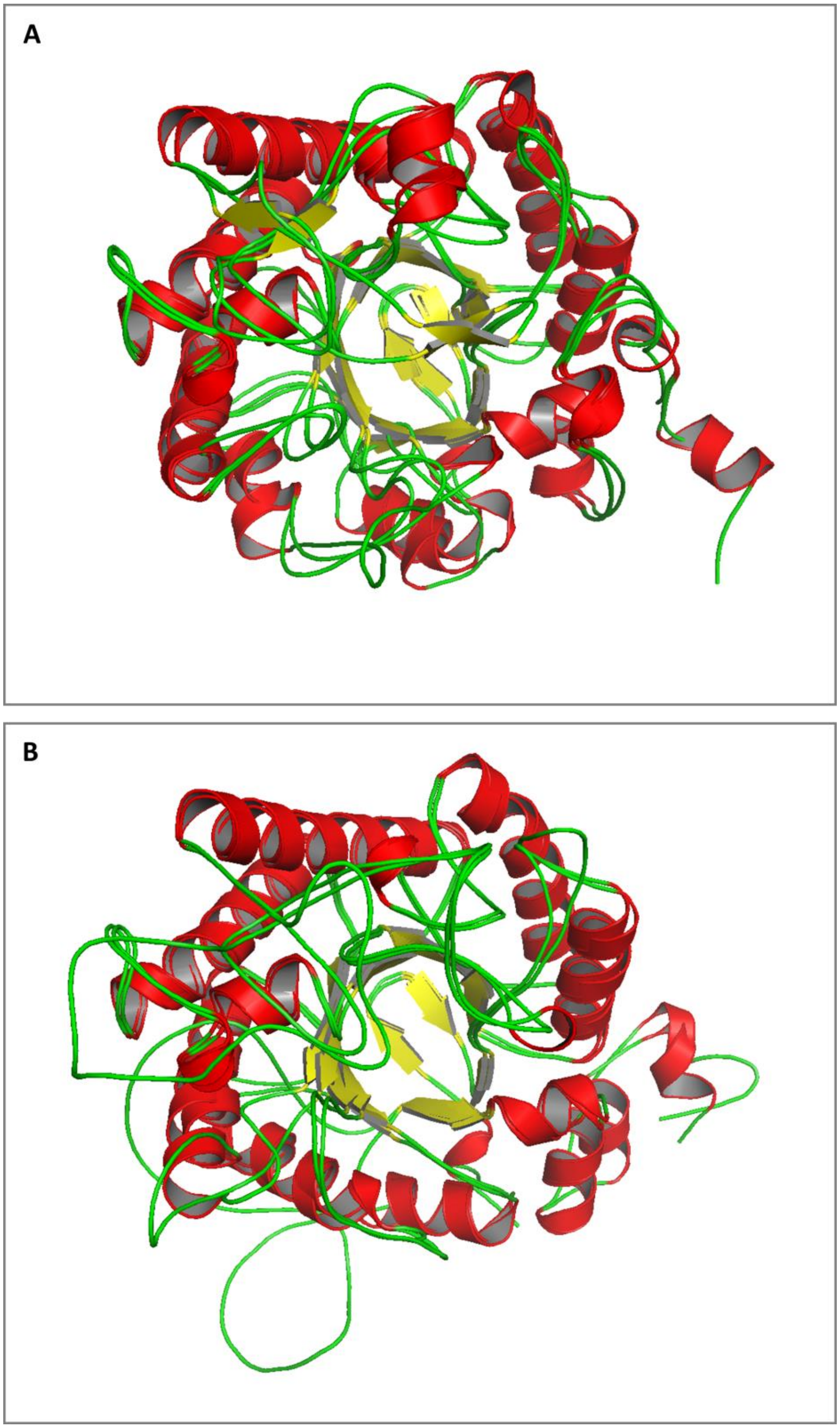

Figure S7. Structural comparison of the modeled Class I (A) and Class II (B) ArOYEs. The alpha helices are displayed in red colour, sheets are displayed in yellow and loops are displayed in green.

A

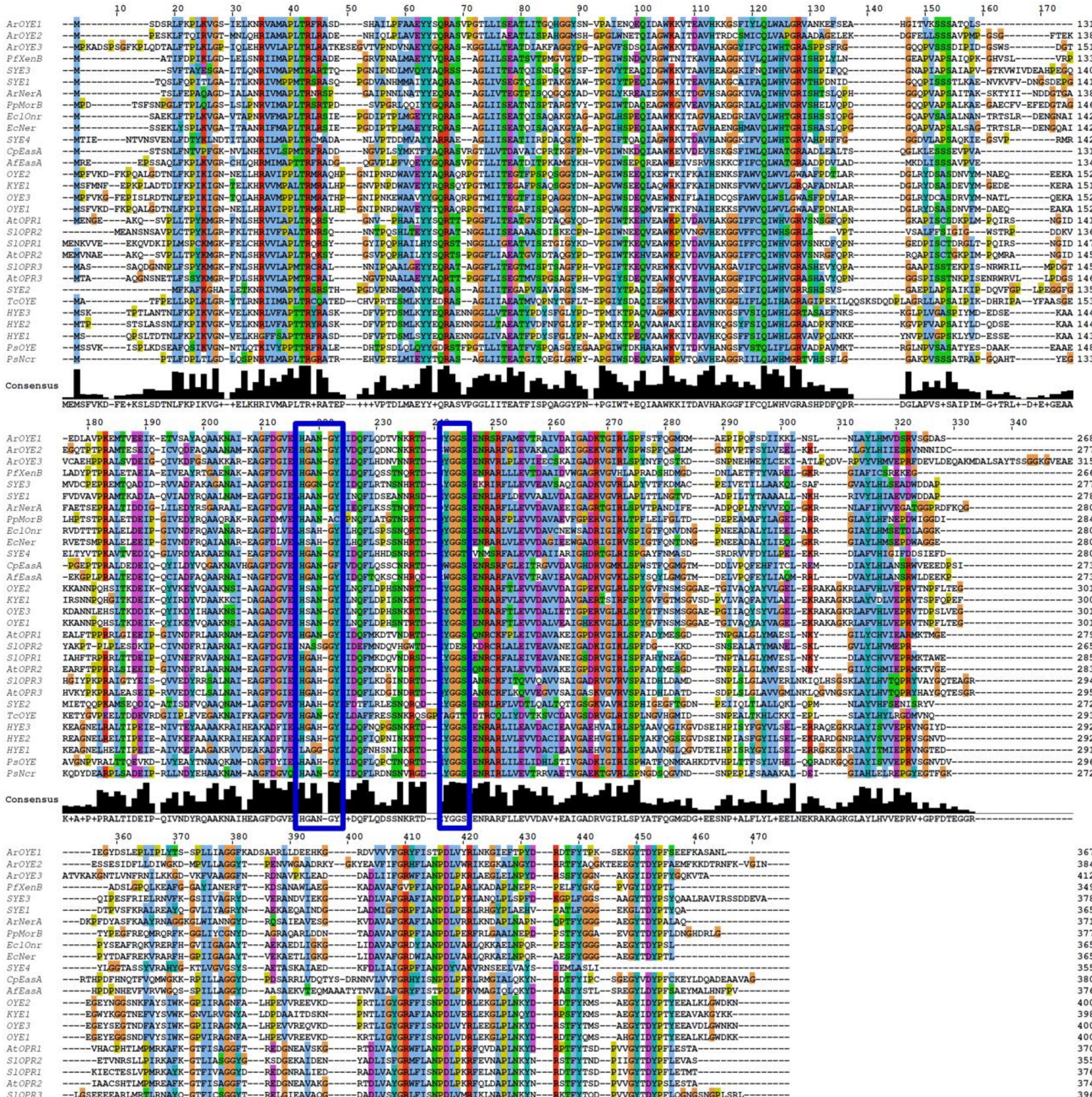

B

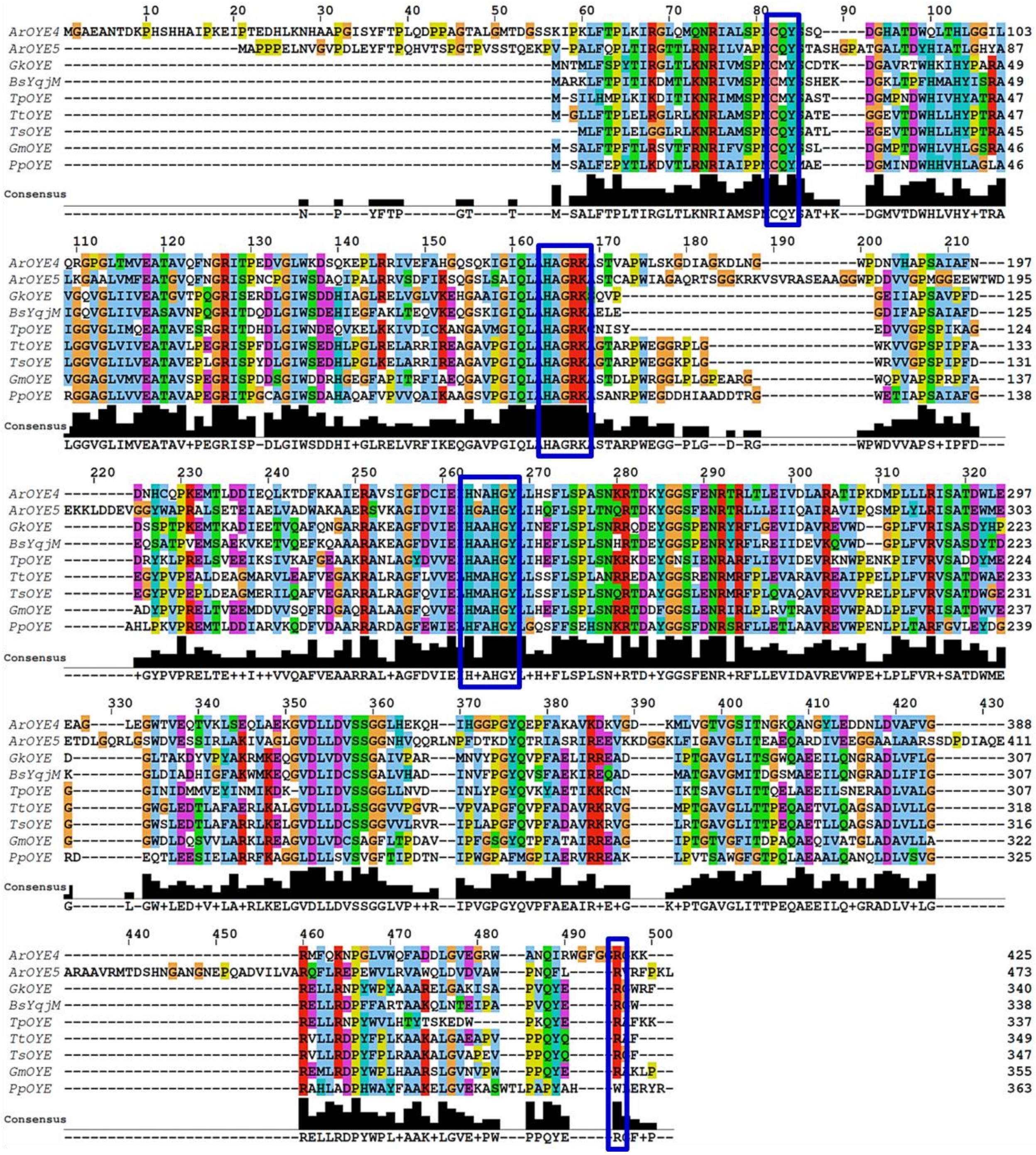

C

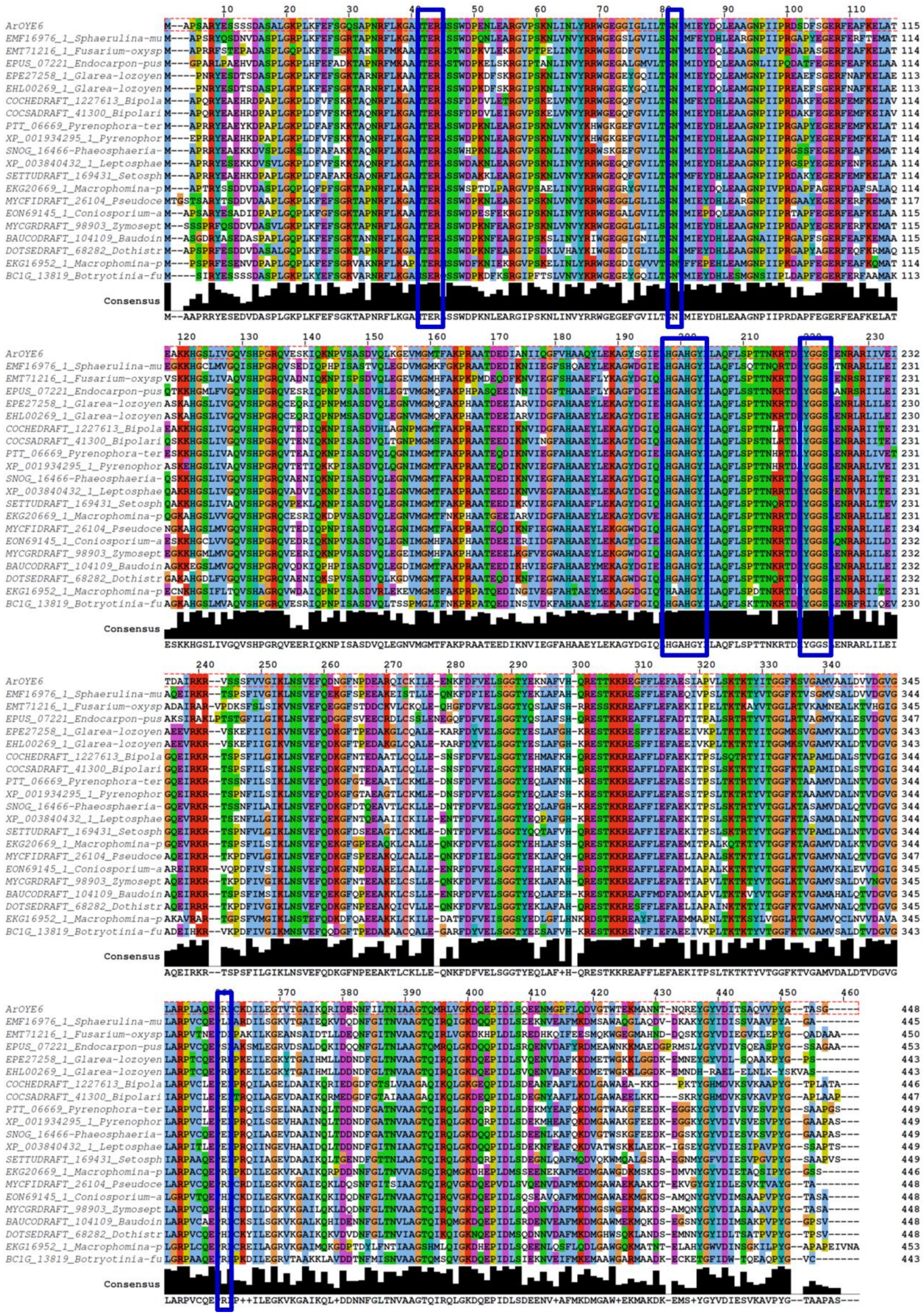
ArOYE6
EMF16976_1_Sphaerulina-mu
EMT71216_1_Fusarium-oxysp
EPUS_07221_Endocarpon-pus
EPE27258_1_Glarea-lozoyen
EHL00269_1_Glarea-lozoyen
COCHEDRAFT_1227613_Bipola
COCSADRAFT_41300_Bipolari
PTT_06669_Pyrenophora-ter
XP_001934295_1_Pyrenophor
SNOG_16466-Phaeosphaeria-
XP_003840432_1_Leptosphae
SETTUDRAFT_169431_Setosph
EKG20669_1_Macrophomina-p
MYCFIDRAFT_26104_Pseudoce
EON69145_1_Coniosporium-a
MYCGRDRAFT_98903_Zymosept
BAUCODRAFT_104109_Baudoin
DOTSEDRAFT_68282_Dothistr
EKG16952_1_Macrophomina-p
BC1G_13819_Botryotinia-fu
Consensus

Figure S8. Class wise multiple sequence alignment of full length OYE proteins. (A) The alignment includes Class I OYEs of *A. rabiei* (ArOYE1-3) and other previously reported OYEs of same Class. (B) Alignment of *A. rabiei* ArOYE4-5 along with previously reported Class II OYEs. (C) Alignment of ArOYE6 along with its top 20 PSI-BLAST hits. All the alignments were generated with the PROMALS3D server using default parameters. The rectangular boxes highlight positions of the conserved active site residues. The consensus sequence is displayed below the alignment.

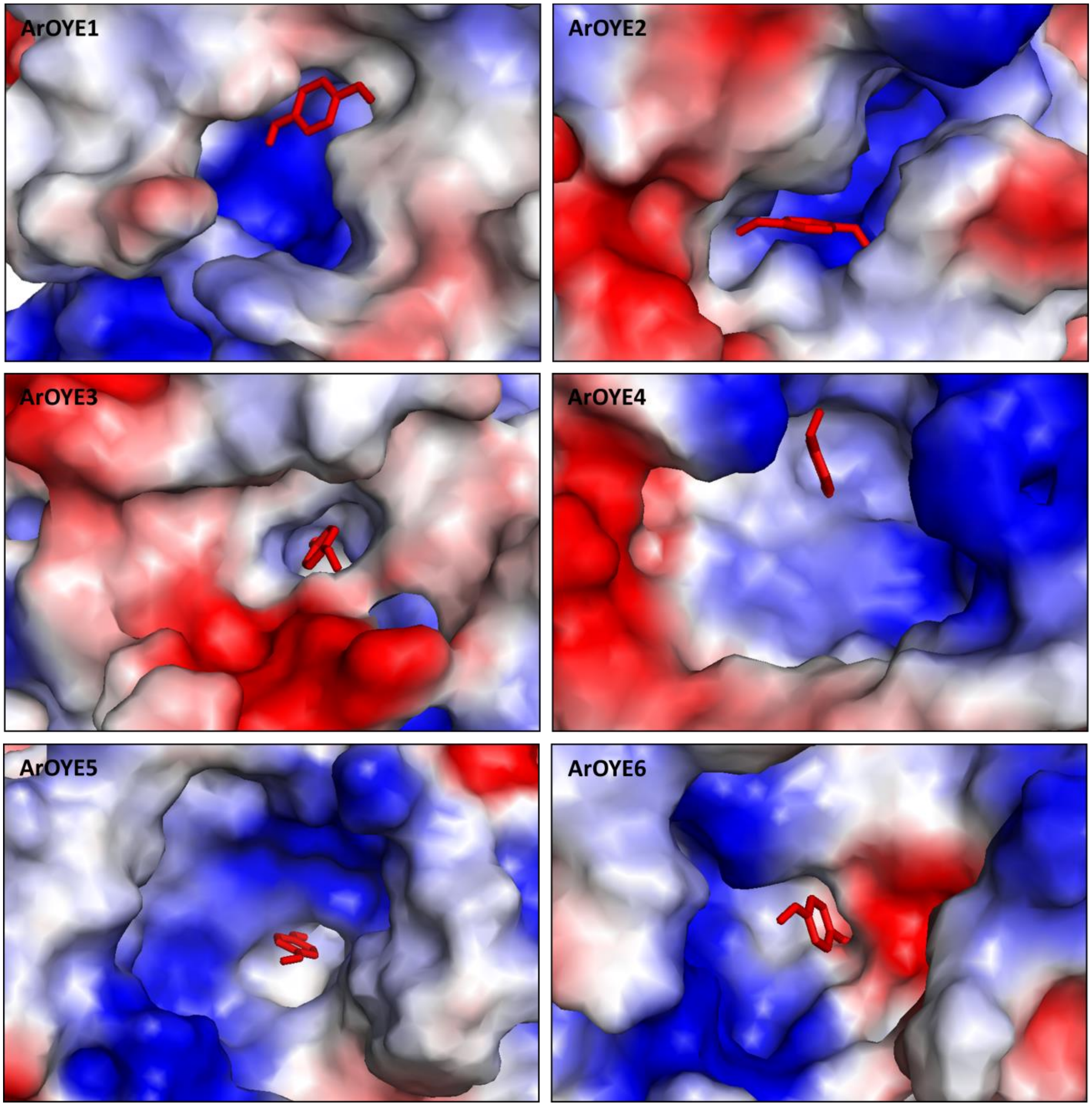

Figure S9. The docking of ArOYEs with *para*-hydroxybenzaldehyde. The ligand *para*-hydroxybenzaldehyde (PHB) was docked into the active site pocket of respective ArOYE. The *para*-hydroxybenzaldehyde is shown in red colour.
